# RNA sequencing least shrew (*Cryptotis parva*) brainstem and gut transcripts following administration of a selective substance P neurokinin NK_1_ receptor agonist and antagonist expands genomics resources for emesis research

**DOI:** 10.3389/fgene.2023.975087

**Published:** 2023-02-14

**Authors:** Kristopher J. L. Irizarry, Weixia Zhong, Yina Sun, Brent A. Kronmiller, Nissar A. Darmani

**Affiliations:** ^1^ College of Veterinary Medicine, Western University of Health Sciences, Pomona, CA, United States; ^2^ Department of Basic Medical Sciences, College of Osteopathic Medicine of the Pacific, Western University of Health Sciences, Pomona, CA, United States; ^3^ Center for Genome Research and Biocomputing, Oregon State University, Corvallis, OR, United States

**Keywords:** genomics, RNA-sequencing, emesis, least shrew, NK_1_ receptor, GR73632, netupitant, signaling

## Abstract

The least shrew is among the subset of animals that are capable of vomiting and therefore serves as a valuable research model for investigating the biochemistry, molecular biology, pharmacology, and genomics of emesis. Both nausea and vomiting are associated with a variety of illnesses (bacterial/viral infections, bulimia, exposure to toxins, gall bladder disease), conditions (pregnancy, motion sickness, emotional stress, overeating) and reactions to drugs (chemotherapeutics, opiates). The severe discomfort and intense fear associated with the stressful symptoms of nausea and emesis are the major reason for patient non-compliance when being treated with cancer chemotherapeutics. Increased understanding of the physiology, pharmacology and pathophysiology underlying vomiting and nausea can accelerate progress for developing new antiemetics. As a major animal model for emesis, expanding genomic knowledge associated with emesis in the least shrew will further enhance the laboratory utility of this model. A key question is which genes mediate emesis, and are they expressed in response to emetics/antiemetics. To elucidate the mediators of emesis, in particular emetic receptors, their downstream signaling pathways, as well as the shared emetic signals, we carried out an RNA sequencing study focused on the central and peripheral emetic loci, the brainstem and gut. Thus, we sequenced RNA extracted from brainstem and gut tissues from different groups of least shrews treated with either a neurokinin NK_1_ receptor selective emetic agonist, GR73632 (5 mg/kg, i.p.), its corresponding selective antagonist netupitant (5 mg/kg, i.p.), a combination of these two agents, *versus* their corresponding vehicle-pretreated controls and drug naïve animals. The resulting sequences were processed using a *de novo* transcriptome assembly and used it to identify orthologs within human, dog, mouse, and ferret gene sets. We compared the least shrew to human and a veterinary species (dog) that may be treated with vomit-inducing chemotherapeutics, and the ferret, another well-established model organism for emesis research. The mouse was included because it does not vomit. In total, we identified a final set of 16,720 least shrew orthologs. We employed comparative genomics analyses as well as gene ontology enrichment, KEGG pathway enrichment and phenotype enrichment to better understand the molecular biology of genes implicated in vomiting.

## Highlights

- Least shrew is a key model organism for investigating emesis at the biochemical level
- Sequencing of Least shrew gut and brainstem provide 16,720 emesis associated transcripts
- Constructed 125 emesis candidate gene set from assembled sequences
- Identified 6,952 one-to-one protein orthologs across shrew, ferret, dog, mouse, and human
- Genomics data produced in this study expands utility of Least shrew as emesis research organism

## 1 Introduction

Emesis research is important because many individuals suffer from vomiting, including human and veterinary cancer patients taking chemotherapeutics. A major research model for emesis is the least shrew, which is investigated to better understand the relationship between gut and brainstem emetic circuits. To date, very few genes in this species have been sequenced, and this has limited genomic approaches to investigate vomiting. The role of this study is to expand genomic resources through targeted RNA sequencing of gut and brain stem in response to emetic/antiemetic agents. By identifying these important transcripts/genes and mapping them to species of interest in the field of vomiting, will enhance the value and application of laboratory animal models of emesis. The least shrew appears to be an optimal emesis model because of its small size, requires small amounts of expensive emetic and antiemetic agents, needs smaller vivarium space, and exhibits sequence homology to humans and veterinary species.

Use of large animals such as cats and dogs for laboratory emesis studies are not only cost ineffective but also socially unacceptable. Smaller vomit competent animals have been validated. Though still large (0.7–2 kg each), ferrets (*Mustela putorius furo*) have often been used. Indeed, ferrets played a key role in both the identification of emetic circuits as well as in the development of serotonin 5-HT_3_-and substance P (SP) neurokinin NK_1_-receptor antagonist antiemetics (Rojas and Slusher, 2015). These antiemetics play a major role in suppression of chemotherapy-induced vomiting (CIV) in both human and veterinary patients receiving cancer chemotherapeutics (Kobrinsky, 1988; Hesketh et al., 2017). The major emetic neurotransmitters involved in CIV include serotonin, SP, and dopamine in both the brainstem emetic nuclei of dorsal vagal complex (area postrema, nucleus tractus solitarius and dorsal motor nucleus of the vagus), as well as in the gastrointestinal tract in the periphery (Darmani et al., 2009; Darmani and Ray, 2009). In the late 1980s a much smaller emesis model (70–100 g) was introduced, the house musk shrew (*Suncus murinus*) (Ueno et al., 1988). In the phylogenetic system shrews are closer to primates than rodents, lagomorphs, and carnivores (Colbert, 1958).

*Cryptotis parva*, the Least shrew, is 10–25 times smaller (adult weighing 4–6 g) than house musk shrews and is found in Central and North America. In the late 1990s our laboratory introduced the least shrew as a new model of emesis (Darmani, 1998). Since then, it has become one of the leading models for revealing diverse post-receptor intracellular emetic signals of vomiting including those following specific activation of dopamine D_2_-, serotonin 5-HT_3_-and SP NK_1_- receptors by their corresponding selective agonists (Zhong et al., 2014b; Zhong et al., 2016; Zhong et al., 2018; Zhong et al., 2019; Belkacemi and Darmani, 2020; Belkacemi et al., 2021). Unlike other emesis models (King, 1990), house musk shrews do not vomit in response to peripheral injection of apomorphine (a non-selective dopamine D_1/2/3/4/5_ receptor agonist) (Ueno et al., 1988; Jenner and Katzenschlager, 2016), whereas ferrets lack an emetic response to intraperitoneal administration of either serotonin or SP (Knox et al., 1993). In contrast, the least shrew is a more versatile model since it robustly vomits not only following intraperitoneal administration of serotonin (Darmani, 1998), dopamine (unpublished findings) or SP (Darmani et al., 2008), but also after peripheral injection of the more selective agonists of 5-HT_3_ (e.g., 2-methylserotonin)-, neurokinin NK_1_ (e.g., GR73632)- and D_2/3_ (e.g., quinpirole, quinelorane, respectively)-receptors. Moreover, least shrews vigorously vomit following apomorphine injection (Darmani et al., 1999; Darmani and Crim, 2005).

Except for humans, no published full nucleotide or amino acid sequences for either the NK_1_ receptor or its endogenous agonist SP is available for the currently used small animal models of vomiting. Using RTPCR, we have partially sequenced approximately a 700 base-pair fragment of the least shrew NK_1_ receptor which has 89%–90% overall sequence identity to humans and chimps (Darmani et al., 2013). In addition, the cDNA for the least shrew SP-producing preprotachkynin-1 (β-PPT1) was cloned and partially sequenced by us and found to be 90% identical to the human sequence, with the SP-producing portion identical to humans (Dey et al., 2010).

RNA sequencing (RNA-seq) enables the construction of transcriptomes from cDNA libraries through massively parallel next-generation DNA sequencing technology (Ozsolak and Milos, 2011). RNA-seq offers advantages over array-based expression studies since unlike nucleotide arrays that require previously known transcript sequence to be encoded on the array to detect transcripts, RNA-seq allows for detection of transcripts without prior knowledge of their sequence. The sequencing data produced from the complete set of RNA molecules enables a wide array of applications including: 1) transcript sequence identification, 2) differential gene expression, 3) biological pathway analysis, and 4) enriched category tests (Han et al., 2015). *De-novo* transcript assembly allows for the construction of cDNA sequences without the need for a reference genome (Moreton et al., 2015). Although *de novo* assembly presents some challenges, such as differentiating between alternative isoforms produced from a single gene and transcripts produced from paralogous genes, the resulting data enables genomics studies to be applied to organisms for which reference genome is not available. The applications of RNA-seq are ever expanding and include investigations of single cell gene-expression, spatial transcriptomics, and RNA structure (Stark et al., 2019). This methodology has been applied to investigations of postoperative nausea and vomiting associated with sevoflourine (Hayase et al., 2016), and more recently has been used to characterize the mechanism associated with antiemetic treatments (Li et al., 2020).

RNA-sequencing has tremendous value in pharmacological research and can aide in elucidating drug targets, downstream mediators of pharmacological therapies as well as genes underlying individual differences in response to drug treatment including variation in efficacy and adverse drug events (Sa et al., 2018). Since 1950, the approval of new US drugs has declined every decade at roughly the same rate (Scannell et al., 2012). Moreover, a peak in drug development in the 1990s and early 2000s has declined to a two-decade low (Berndt et al., 2015). The potential impact of RNA-seq on the identification, development and commercialization of novel pharmacological therapies is an important application. Specifically, the information contained in genomics data sets obtained from patient transcriptome data, cell-line associated transcriptome data, and model organism transcriptome data may expedite discovery of drug-disease associations and characterization of candidate drug targets (Kwon et al., 2019).

**FIGURE 1:**
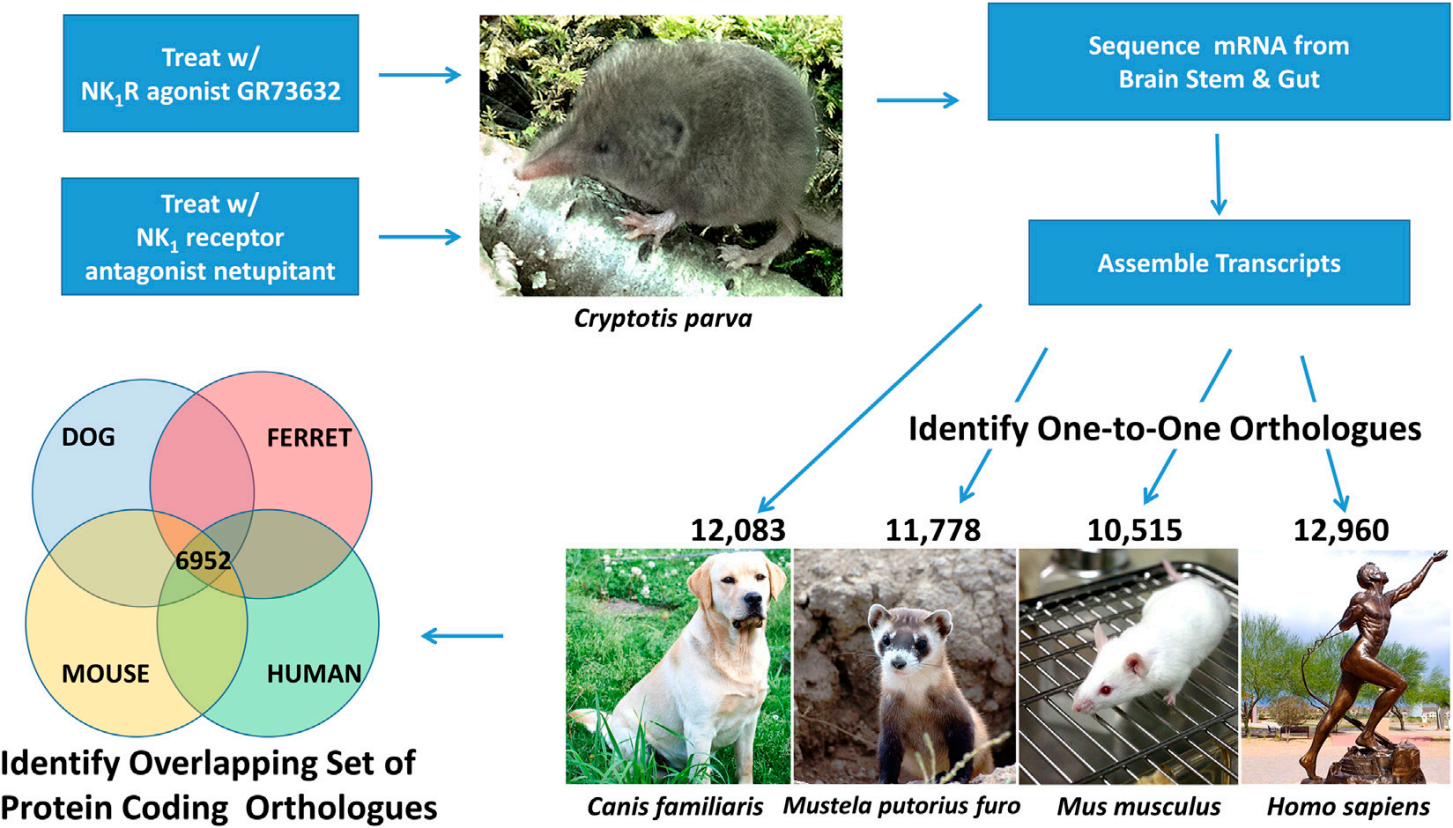
Overview of RNA Sequencing Study Design. This genomics study investigated the transcripts induced in the brain stem and gut following least shrew (*Cryptotis parva*) treatment with an NK_1_R agonist (GR73632) and an NK_1_R antagonist (netupitant). Brain stem and gut transcripts were assembled from overlapping RNA sequencing reads and one-to-one orthologs between shrew and dog (*Canis familiaris*), ferret (*Mustela putorius furo*), mouse (*Mus musculus*), and human (*Homo sapiens*). The resulting ortholgous sequences were used to identify a set of 6,952 shared orthologs across all five species (shrew, dog, ferret, mouse, human). Images of each species were acquired from the open source image repository wikimedia.org (https://commons.wikimedia.org): dog (Afra_008.jpg), ferret (Iltisfrettchen.jpg), mouse (Lab_mouse_mg_3158.jpg), human (Bronze_anatomical_figure,_Europe_Wellcome_L0058953.jpg), shrew (Shrew1opt.jpg).

Our rationale for applying RNA-seq to the least shrew, *Cryptotis parva*, was to identify the transcriptomes associated with: a) the regions of the brainstem implicated in the control of emesis, as well as b) the tissues and cells underlying emesis in the gut (Figure 1). Because the least shrew is an optimal model for investigating the pharmacology of vomiting, the construction of relevant transcripts can help guide biochemically driven, pharmacological studies. Here we report the construction and analysis of the transcriptomes identified in the brainstem and gut under treatments with an agonist and antagonist of the SP neurokinin NK_1_ receptor. Our genomics data sets and resulting analyses provide resources for further investigations into emetic/antiemetic pharmacology in the least shrew.

## 2 Materials and methods

### 2.1 Animals

Adult least shrews from the Western University of Health Sciences Animal Facilities colony were housed in groups of 5–10 on a 14:10 light:dark cycle and were fed and watered *ad libitum*. The experimental shrews were between 45 and 60 days old and weighed 4–6 g. Animal experiments were conducted in accordance with the principles and procedures of the National Institutes of Health Guide for the Care and Use of Laboratory Animals Protocol number R20IACUC018). All protocols were approved by the Institutional Animal Care and Use Committee of Western University of Health Sciences. All efforts were made to minimize animals’ suffering and to reduce the number of animals used in the experiments. Each shrew was used only once and following completion of each experiment the tested shrews were euthanized with 32% isoflurane *via* inhalation.

### 2.2 Chemicals

The following drugs were used in the present study: the NK_1_R agonist GR73632 (Sakurada et al., 1999) was purchased from Tocris, Minneapolis, MN. The NK_1_ receptor antagonist netupitant was kindly provided by Helsinn HealthCare (Lugano, Switzerland). GR73632 was dissolved in distilled water and netupitant was dissolved in a 1:1:18 solution of emulphorTM, ethanol and saline. All drugs were administered at a volume of 0.1 ml/10 g of body weight.

### 2.3 Behavioral emesis studies

On the day of the experimentation least shrews were brought from the animal facility, separated into clean individual cages (20 × 18 × 21 cm) lined with wood chippings, and were allowed to adapt for at least 2 hours (h). Daily food was withheld 2 h prior to the start of the experiment but shrews were given four mealworms each prior to emetogen injection, to aid in identifying wet vomits as described previously (Darmani, 1998).

We have earlier demonstrated that a 5 mg/kg (i.p.) injection of the brain penetrating selective NK_1_ receptor agonist GR73632 produces a robust frequency of vomits in all tested animals (Darmani et al., 2008). We have also shown that pretreatment with the selective NK_1_ receptor antagonist netupitant suppresses the GR73632 (5 mg/kg, i.p.)-evoked vomiting in a dose-dependent manner with complete protection in all tested shrews at 10 mg/kg (Zhong et al., 2019). In the latter study, the 5 mg/kg dose of netupitant protected 85% of shrew from vomiting. To minimize possible effects of netupitant on mRNA expression by itself, we chose its lower effective antiemetic dose (5 mg/kg) for the current drug interaction studies. One animal in the GR73632 + netupitant treatment group vomited and this animal was not included in our data analysis. Thus, different groups of shrews were treated with: i) at 0 min with netupitant vehicle (i.p.) and 30 min later with GR73632 vehicle (i.p.) (*n* = 5), ii) at 0 min with netupitant vehicle (i.p.) and 30 min later with GR73632 (5 mg/kg, i.p.) (*n* = 5), iii) at 0 min with netupitant (5 mg/kg, i.p.) and 30 min later with GR73632 vehicle (i.p.) (*n* = 5); iv) at 0 min with netupitant (5 mg/kg, i.p) and 30 min later with GR73632 (5 mg/kg, i.p.) (*n* = 5); and v) no treatment (i.e. drug naïve) (*n* = 5). Thirty minutes following the last injection, shrew brainstems and gut (jejunum, taken as a 1 cm length cut, 2 cm below the gastric-intestinal junction were cleaned individually with saline (37 ^O^C)) were rapidly dissected and frozen and kept at −80 OC until analysis. At the behavioral level all netupitant-vehicle-pretreated shrews that had received GR73632 vomited. No other treatment group exhibited emesis.

### 2.4 RNA preps and sequencing

RNA samples were sequenced at the Oregon State University Center for Genome Research and Biocomputing (OSU CGRB). Tissue samples of five gut samples and five brainstem samples with three replicates each (30 total) were sent to OSU CGRB. Tissues were extracted using the Zymo Direct-zol RNA MiniPrep with TRI-Reagent kit and quantified using a Qubit fluorometer and Agilent Bioanalyzer 2,100. Extracted RNA sequencing libraries were prepared using the Wafergen RNA kit and quality controlled by qPCR. Samples were sequenced on a 100bp paired end run on an Illumina HiSeq 3,000.

### 2.5 *de novo* transcriptome assembly

Trimmomatic v0.33 (run with parameters: PE ILLUMINACLIP:Adapters.fa:2:30:10 SLIDINGWINDOW:4:30 LEADING:10 TRAILING:10 MINLEN:36) (Bolger et al., 2014) was used for quality control and for adapter trimming raw sequences. Raw sequences from the 15 brainstem replicates and 15 gut replicates were pooled and assembled into a single *de novo* transcriptome assembly using Trinity v2.4.0 (run with parameters: -seqType fq-max_memory 250G-CPU 30) (Grabherr et al., 2011). This pooled *de novo* transcriptome was used as the reference transcriptome for downstream expression and orthology analysis. The Trinity transcript quantification method (Trinity v2.4.0) (https://github.com/trinityrnaseq/trinityrnaseq/wiki/Trinity-Transcript-Quantification) was used to estimate transcript abundance: individual sequences from each replicate for both brainstem and gut samples were aligned using bowtie2 (Langmead and Salzberg, 2012) to the pooled *de novo* reference transcriptome and counted using RSEM (Li and Dewey, 2011) (run with parameters: -seqType fq-est_method RSEM-aln_method bowtie2 -trinity_mode). BAM files were manipulated with SAMtools (Li et al., 2009).

### 2.6 Orthology analysis

Orthologous transcripts were identified between the *de novo* assembled shrew transcriptome and dog, ferret, mouse, and human transcript sequences from the Ensembl data repository. Basic Local Alignment Search Tool (ncbi-blast-2.10.0+) was used to create blast databases for each species (shrew, ferret, dog, mouse, human). Each species’ set of genes was aligned *via* BLASTN (Altschul et al., 1990) to the assembled shrew transcriptome. Orthology was determined using a blast-back approach and custom perl scripts.

### 2.7 Identification of shrew transcripts having human/mouse/dog/ferret orthologs

**FIGURE 2:**
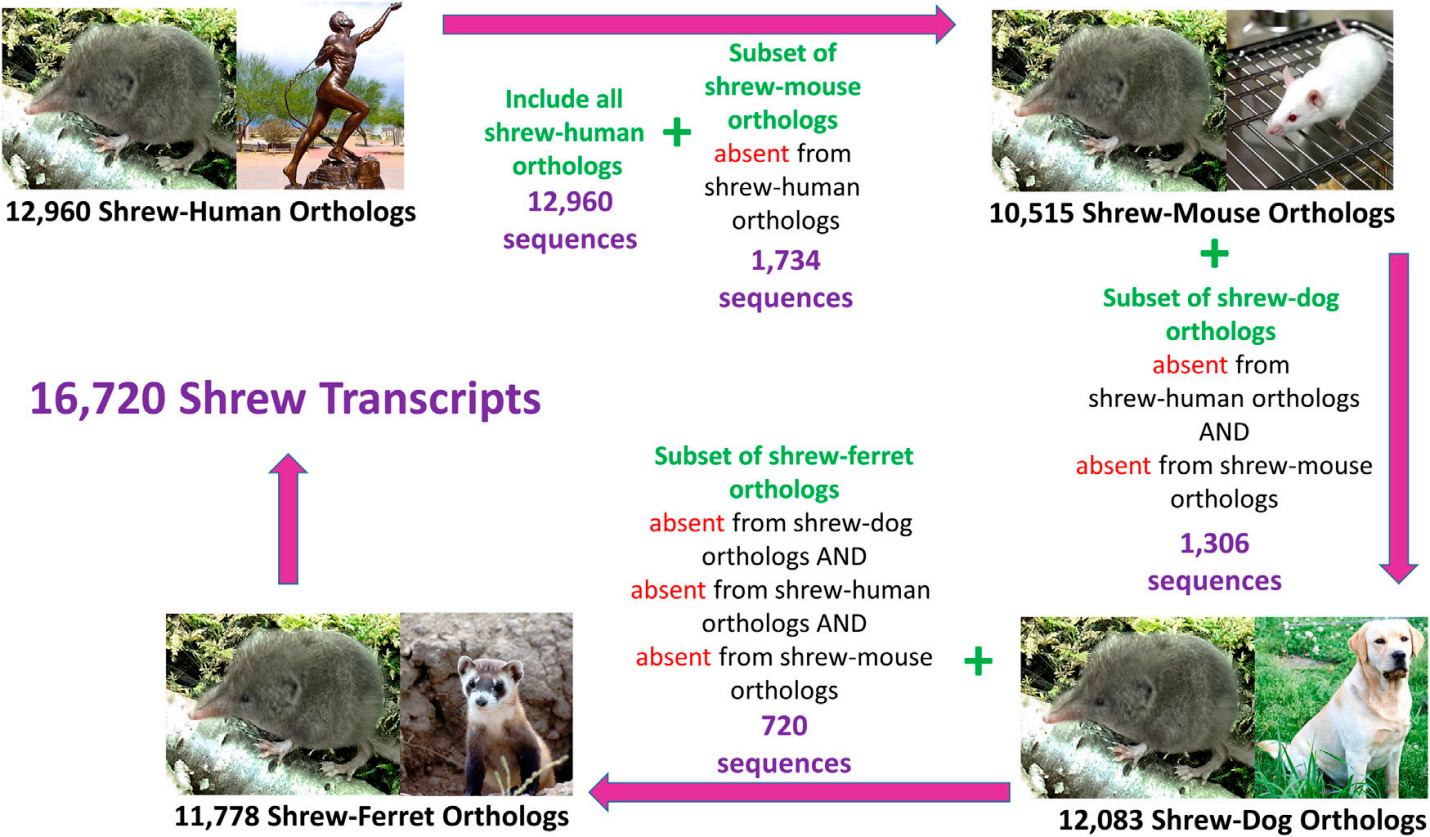
Conceptual strategy for identifying Non-Redundant Orthologs Across Species. One to one orthologs between the least shrew transcript sequences and other species ranged from 10,515 (mouse) to 12,965 (human). Some transcripts were identified in one species, but not in the other species, a cumulative set of shrew transcripts containing 16,720 sequences was compiled that represents the greatest number of shrew orthologues from the four species. Another set, called “shared” transcripts (7,339) was extracted which contained a one-to-one orthologue from each of the four species. Images of each species were acquired from the open source image repository wikimedia.org (https://commons.wikimedia.org): dog (Afra_008.jpg), ferret (Iltisfrettchen.jpg), mouse (Lab_mouse_mg_3158.jpg), human (Bronze_anatomical_figure,_Europe_Wellcome_L0058953.jpg), shrew (Shrew1opt.jpg).

We identified a non-redundant set of shrew transcript sequences which maximized the number of shrew transcript sequences across the set of human, mouse, dog, and ferret orthologs (Figure 2). Specifically, we identified the largest group of shrew transcript sequences that contained human ortholog sequences. Of the remaining shrew transcripts that did not contain human orthologs, shrew orthologs of mouse genes were identified. Shrew sequences that did not contain mouse or human orthologs were used to identify sequences orthologous to dog sequences. Finally, the shrew sequences without orthologs in human, mouse and dog were used to identify shrew orthologs to ferret. The resulting set of mapped orthologs were combined to produce the final non-redundant ortholog transcript set.

### 2.8 Characterization of shrew transcript length

The set of orthologous shrew sequences was characterized using the Maria open source database server. Transcript sequences were loaded into the database and queries were performed to determine minimum, maximum, average and total sequence length. Supplementally, individual queries were performed to characterize the distribution of transcript lengths.

### 2.9 Identification of shrew transcripts implicated in emesis

A set of candidate emesis transcripts were identified from among the orthologous sequences. These candidate sequences were identified using published peer-reviewed papers that identify genes implicated in emesis. Because many genes are expressed in a variety of tissues, we leveraged the complete set of sequencing reads to build our consensus sequences. Future work will include a comprehensive analysis of the tissue specific data to better understand the role tissue specific expression plays in this process.

### 2.10 Functional genomic analysis of candidate emesis gene set

The set of candidate emesis genes were analyzed using the Database for Annotation, Visualization, and Integrated Discovery (DAVID) v6.8. Human official gene symbols for the 128 emesis candidate genes were uploaded to the DAVID database and used to identify genes associated with human diseases contained within the Online Mendelian Inheritance in Man (OMIM) database. Additional analyses included identifying enriched a) gene ontology annotations, b) enriched KEGG pathways, c) enriched tissue expression from the UniProt Tissue Expression database, and d) enriched interacting proteins from the Database of Protein Interactions (DIP).

### 2.11 Protein coding orthologs across shrew/human/mouse/dog/ferret

One-to-one orthologs across shrew, ferret, dog, mouse, and human were identified using a relational database containing tables for shrew-to-ferret, shrew-to-dog, shrew-to-mouse, and shrew-to-human one-to-one orthologs. Queries were performed across these tables using SQL statements to identify the set of protein coding genes that were present in all five species.

### 2.12 Characterization of identity between least shrew and other species' sequences

Basic Local Alignment Search Tool (ncbi-blast-2.10.0+) was used to create blast databases for each species (shrew, ferret, dog, mouse, human). Biomart (https://www.ensembl.org/biomart/martview/) (Yates et al., 2020) was used to download the cDNA sequences from ferret, dog, mouse and human. Fasta files were used as input to create blast databases for each non-shrew species. Blastn was used to determine nucleotide identity with the following parameters: max_target_seqs 1,000 -max_hsps 1,000 -outfmt 6. Blastx was used to compare shrew transcript sequences to translated versions of the cDNA from the other four species. Statistical significance of the average identity between pairs of species (shrew-dog; shrew-ferret; shrew-mouse; shrew-human) was calculated using an online z-score calculator for two population proportions (Social Science Statistics, https://www.socscistatistics.com/tests/ztest/default2.aspx) with an alpha = 0.05. This calculation was repeated for both nucleotide identity and amino acid identity. Heatmaps showing percent identity of the subset of candidate emesis genes intersected with the 6,999 nucleotide and 6,952 amino acid conserved orthologous sequences were created with R using the heatmap package.

A set of emesis pathway genes that have been identified in the least shrew to date through biochemical analyses were compiled and used to identify the set of those pathway members from among our set of 16,720 orthologous genes. Considerable research into the biochemistry and pharmacology of emesis has used the least shrew as a model organism, and therefore the results identifying the components of the pathway have been identified in least shrews. These studies have guided our work very closely, and we have compiled a comprehensive list of 39 pathway members and compared the sequence relationships between our least shrew data and the existing transcripts publicly available for human, dog, mouse, ferret, and a shrew in the genus *Sorex* (*Sorex araneus*).

Online sequence identity search performed using Ensembl release 107 from July 2022 (https://uswest.ensembl.org/index.html). Web-based blast search was performed using Ensembl against the following species: human, mouse (CLC57BL6), dog, ferret, shrew. Best hit from each species was identified and percent identity, E-value, and transcript identifier of top hit was recorded. Individual blast results for each pathway associated gene *versus* each of the targeted species was downloaded. A table was compiled from the data (Howe et al., 2021). For the analysis of the emesis pathway members and identification of top scoring transcripts from human, dog, mouse, ferret, and shrew (Sorex) the Ensembl genome database (version 107) was used. The parameters for blast included a limit of reporting 100 hits, with a maximum of 100 high scoring pairs, E-value chtoff = 10, word size = 11, match score = 1, mismatch score = −3, gap opening penalty = 2, gap extension penalty = 2. Low complexity regions were filtered, and query sequences were repeat masked.

### 2.13 Phenotype enrichment of most conserved protein coding orthologs

The 6,952 conserved protein coding genes were ordered by decreasing percent identity between the shrew sequences and human sequences produced from the blastx output. The ordered set of sequences was partitioned into ten subsets, with the first most conserved 697 sequences going into the first partition and the remaining nine partitions containing 695 sequences each such that sequences in each partition were more conserved than the sequences in the subsequent partitions. The resulting deciles of the 6,952 most conserved one-to-one protein coding orthologs across shrew, ferret, dog, mouse, and human were assessed for phenotype enrichment analysis using the Model Organism Phenotype Enrichment toolkit (http://evol.nhri.org.tw/phenome2/). This analysis was performed using “human” as the species of the gene list and mouse phenotypes as the phenotypes to be analyzed. The choice to use human gene symbols was made because human gene symbols are well recognized by many analysis platforms compared to gene symbols from species such as the ferret or dog. The phenotype enrichment was initiated for each decile of the 6,952 genes *versus* the remaining genes in the human genome. All levels of phenotype categories were selected for the analysis corresponding to levels 2 through 16. The analysis was conducted under the alternative hypothesis that the ∼695 genes in each gene set were enriched for specific phenotypes compared to the remaining genes in the rest of the genome using Fisher’s Exact Test.

### 2.14 KEGG pathway enrichment of most conserved protein coding orthologs

The 6,952 conserved protein coding genes were ordered by decreasing percent identity between the shrew sequences and human sequences produced from the blastx output and partitioned into ten subsets, with the first containing 697 sequences and the remaining nine partitions containing 695 sequences each. KEGG pathway enrichment was performed using the Database for Annotation, Visualization and Integrated Discovery (DAVID) v6.8 (DAVID bioinformatics database, https://david.ncifcrf.gov/) (Huang da et al., 2009a; b).

## 3 Result

### 3.1 Identification and characterization of orthologous shrew transcript sequences

**FIGURE 3:**
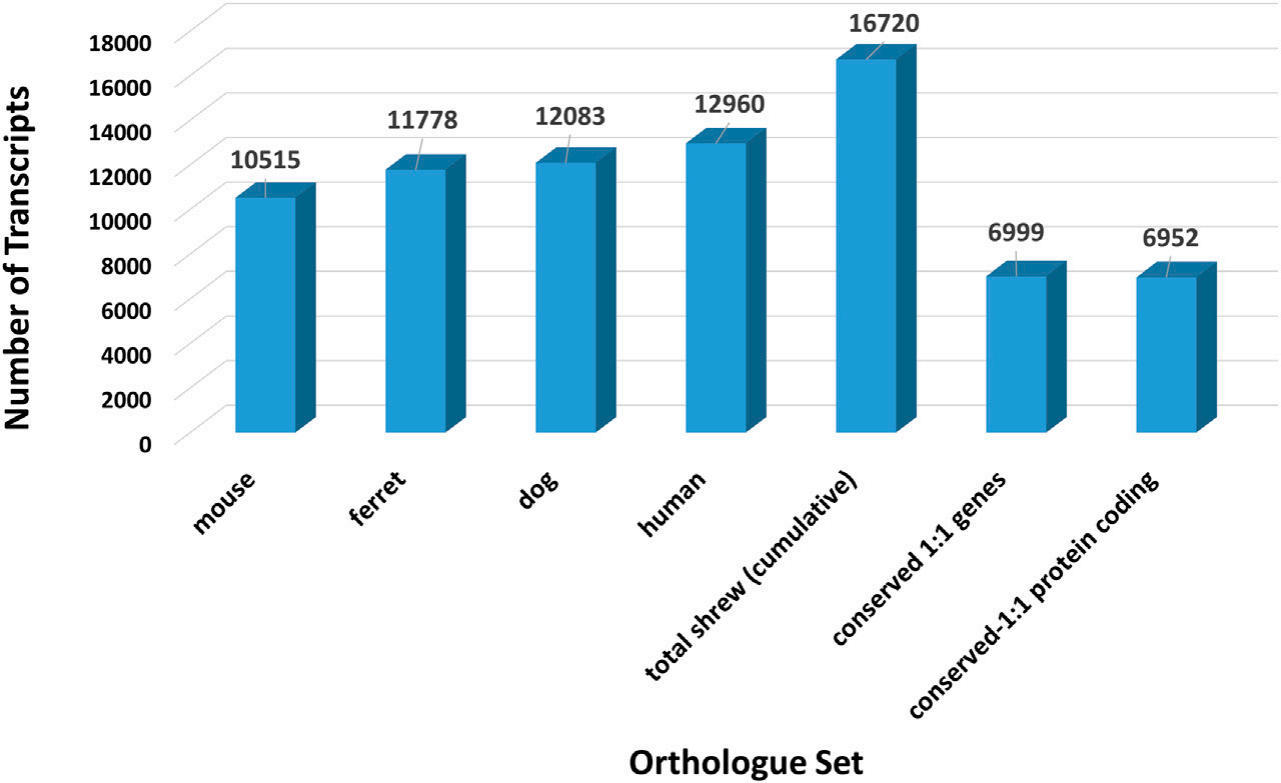
Number of orthologous transcript sequences across species and shared among species. Total number of identified ortholgous sequences between the initial set of transcript fasta sequences assembled from the shrew (*Cryptotis parva*) brain stem and gut RNA sequencing reads and nucleotide sequences from dog (*Canis familiaris*), ferret (*Mustela putorius furo*), mouse (*Mus musculus*), and human (*Homo sapiens*). The complete set of orthologous transcript sequences identified in shrew by assembling transcripts across dog, ferret, mouse and human (illustrated in Figure 2) is shown for comparison purposes. Conserved genes and protein coding genes across all five species (shrew, dog, ferret, mouse, human).

RNA sequencing produced 38 billion base pairs of brainstem and 37.2 billion base pairs of gut. This data was used to assemble a *de novo* reference transcriptome (contigs 623,905, assembly length 241,685,294 bp, average length 387.4 bp, N50 385). To identify a set of representative gene transcripts from among these assembled fasta sequences, we selected one-to-one orthologs between the shrew sequences and human, dog, mouse, and ferret sequences. To identify the largest set of shrew orthologous transcripts, sequences were mapped to orthologs across human, mouse, dog, and ferret Supplemental File S10 (Figure 2). Shrew transcripts mapping to multiple species were associated with the human version of the gene, if possible. The remaining shrew sequences were preferentially mapped to mouse, then dog, and finally ferret orthologs that were not already identified in the human orthologs. A total of 16,720 orthologous shrew transcripts were identified among the total orthologs mapped across each species (Figure 3).

**FIGURE 4:**
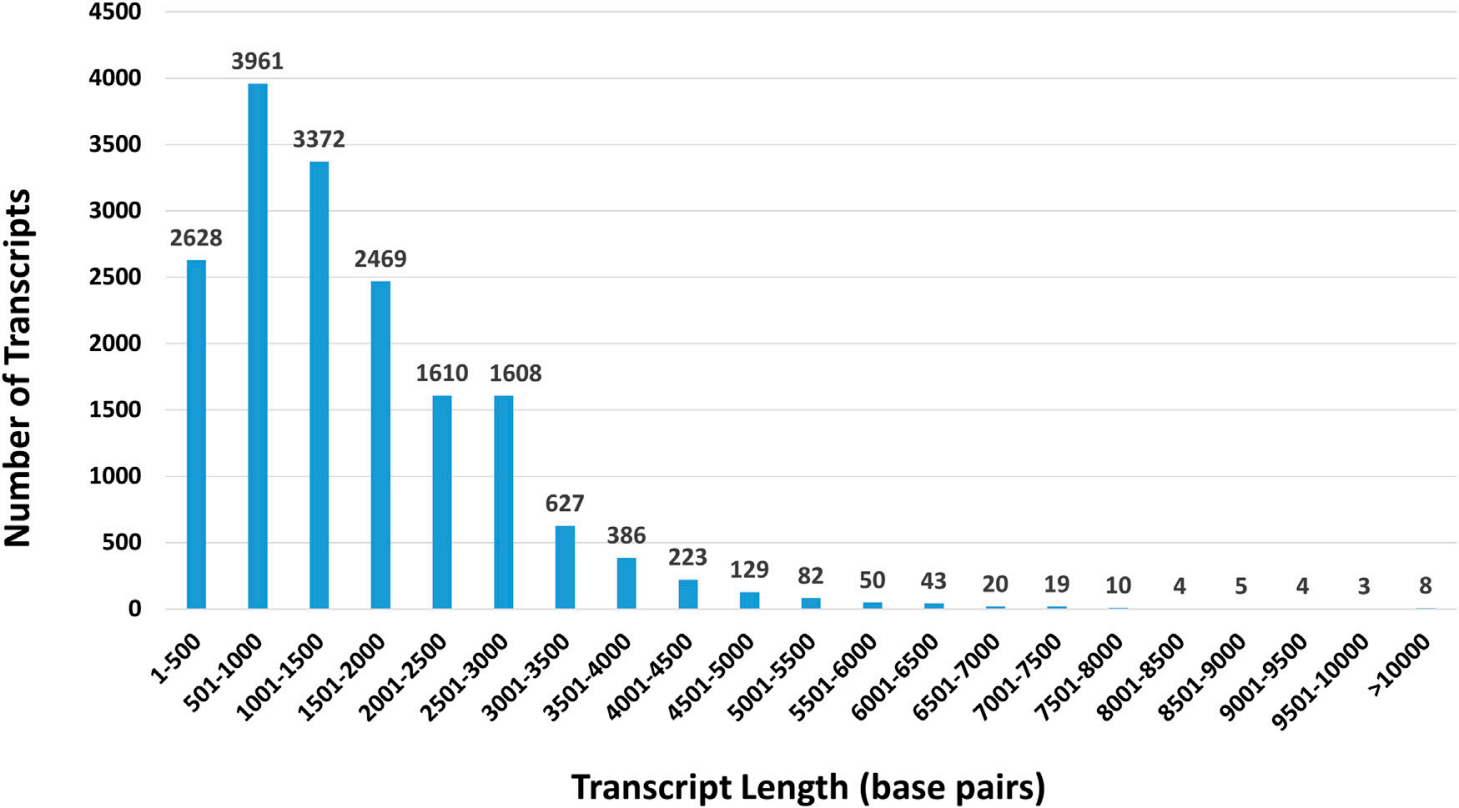
Distribution of nucleotide lengths across 16,720 orthologous shrew transcript sequences. The distribution of sequence lengths among the complete set of 16,720 shrew transcripts were assessed. Sequences ranged from 201 bp to 15,430 bp. Most shrew transcripts were 4,000 bp or less in length. Fewer than 500 sequences were longer than 5,000 bp.

The distribution of nucleotide lengths across the orthologous shrew genes are shown in Figure 4. The average nucleotide length of shrew transcripts was 1,518 bp with a standard deviation of 1,146 bp. Many of the shortest transcripts we identified are under 500 bp in length. In fact, we identified 954 transcripts under 300 bp and 2,622 transcripts under 500 bp in length. For example, the shortest transcripts we identified are 201 bp long and we identified 17 transcripts that were exactly 201 bp.

A representative set of six of these short 201 nucleotide shrew transcripts corresponding to ferret, dog, mouse and human orthologs were compared to the length of the corresponding orthologous transcript (Table 1). Each of these short transcripts was considerably shorter than the corresponding orthologous transcript sequence from the other species. The shortest orthologous transcript sequence was 488 bp (TPM1 from ferret, ENSMPUT00000000657) while the three other 201 nucleotide transcripts had orthologous transcripts which were 795 bp (TLCD2 from ferret, ENSMPUT00000013334), 861 bp (OTUD6A from dog, ENSCAFT00000026526) and 920 bp (NAA20 from human, ENST00000310450). The two longest orthologous transcripts mapping to the set of six short shrew transcripts were 5,000 bp (Fbxw8 from mouse, ENSMUST00000049474) and 4,704 bp (TPM1 from ferret, ENSMUST00000049474). Based on these comparisons, our short shrew transcript sequences are most likely incomplete assemblies of larger transcripts.

**TABLE 1:** Representative examples of shrew transcript sequences. A set of representative short transcript sequences identified in the set of 16,720 shrew transcripts. The corresponding orthologous sequence from the corresponding species is listed along with the length of the sequence in that species. Gene symbols and corresponding gene names: Fbxw8 (F-Box And WD-40 Domain-Containing Protein 8); TPM1 (Tropomyosin 1); (WIPF1) WAS/WASL-interacting protein family member 1; (NAA20) N-alpha-acetyltransferase; (TLCD2) TLC domain-containing protein 2; (OTUD6A) OTU domain-containing protein 6A.

Shrew cDNA identifier	Symbol	Species	Transcript identifier	Transcript length
TRINITY_DN308915_c4_g1	Fbxw8	mouse	ENSMUST00000049474	5,000 bp
TRINITY_DN317929_c4_g1	TPM1	ferret	ENSMPUT00000000657	4,704 bp
TRINITY_DN323061_c3_g2	WIPF1	human	ENST00000455428	488 bp
TRINITY_DN306984_c4_g3	NAA20	human	ENST00000310450	920 bp
TRINITY_DN326659_c1_g1	TLCD2	Ferret	ENSMPUT00000013334	795 bp
TRINITY_DN312853_c1_g1	OTUD6A	dog	ENSCAFT00000026526	861 bp

The largest transcript in our data set (TRINITY_DN328899_c3_g1) is 15,430 bp and encodes apolipoprotein B for which the human ortholog (UniProtKB - P04114) contains 4,563 amino acids. The second largest transcript we identified (TRINITY_DN328883_c1_g1) is 12,275 bp and encodes LDL receptor related protein 1, for which the human version of this protein (UniProtKB - Q07954) is 4,544 amino acids in length. The third largest transcript (TRINITY_DN328880_c2_g1) is 12,240 bp in length and encodes the shrew version of the 7,570 amino acid human protein dystonin (UniProtKB - Q03001). Finally, the fourth largest shrew transcript (TRINITY_DN328901_c5_g1) is 11,679 bp long and encodes an ortholog of human microtubule associated protein 1B (UniProtKB - P46821) that is 2,468 amino acids in length. These comparisons suggest that the long shrew transcript sequences correspond to comparably long orthologous transcripts from orthologous species. In order to assess how well these new shrew transcript sequences compared to previously described transcripts, several important shrew genes were aligned to assembled transcripts to show sequence comparisons.

### 3.2 Comparison of tachykinin precursor one transcript sequences

The least shrew tachykinin precursor 1 mRNA sequence was previously reported and deposited into NCBI (JUL-2016) with the accession number FJ696706. The sequence length was listed as 215 bp. The sequence we identified in this study for the tachykinin precursor is 508 bp. The two sequences were compared using pairwise blast (Supplemental File 1). The RNA-seq version of the transcript matched 100% with 215 out of 215 nucleotides aligning. The e-value for this match is 1e-115.

### 3.3 Comparison of brain derived neurotrophic factor transcript sequences

Pairwise BLAST alignment between the NCBI deposited Brain Derived Neurotrophic Factor sequence and the RNA-seq version of the sequence (Supplemental File 2). Although the RNA-seq version of the gene is larger than the originally deposited sequence, the overlapping regions matched 100% with no gaps. BDNF transcripts overlap 564 nucleotides for which the alignment contains 0 gaps and is 100% identical.

### 3.4 Comparison of neurokinin receptor 1 transcript sequences

Pairwise BLAST alignment between the NCBI deposited Neurokinin Receptor 1 sequence (Query sequence) and the RNA-seq version of the sequence (Subject sequence). The RNA-seq version of the gene is longer than the NCBI deposited sequence. The region of overlap between the two sequences exhibits 100% identity with no gaps over 602 nucleotides (Supplemental File 3).

### 3.5 Candidate genes associated with emesis

A set of 125 emesis-candidate genes were identified among the transcripts we generated in this study. The candidate set was hand curated from the RNA sequencing data and our previous studies relating to least shrew emesis and the downstream intracellular emetic signaling molecules post NK_1_ neurokinin receptors (Supplemental File 4).

### 3.6 Functional analysis of shrew candidate emesis genes

To better understand the biological associations within the set of shrew candidate emesis genes, functional genomics analysis was performed to identify annotations capable of providing insight into the roles of these genes. The first analysis that was performed identified the set of human diseases associated with members of these shrew candidate genes (Supplemental File 5). This analysis utilizes the Online Mendelian Inheritance in Man (OMIM) database which curates and provides resources to investigate the relationships between genes and diseases in humans.

The results include a number of diseases associated with neurological and sensory deficits including deafness, blindness, dopamine beta-hydroxylase deficiency, as well as neuropsychiatric disorders including obsessive compulsive disorder and schizophrenia. A variety of neuronal synaptic signaling genes are associated with diseases including members of the adenylate cyclase gene family, calcium voltage-gated channels, neurotransmitter receptors, neurotransmitter biosynthetic enzymes, and intracellular signaling molecules such as phospholipase and protein kinase. The discovery of these gene-disease associations in the least shrew expands the utility of the shrew as a model for psychiatric and neurological disorders, including vomiting and nausea.

In order to gain a better understanding of the functional genomic roles for these candidate emesis genes, we employed a gene ontology enrichment analysis (GOA). Because the goal of GOA is to identify constellations of genes that all share the same ontological annotations, this analysis facilitates a systematic dissection of the genes into groups associated with specific biological processes (Table 2 contains most significant results, Supplemental File six contains all significant results), molecular functions (Table 3 contains most significant results, Supplemental File seven contains all significant results) and cellular locations (Table 4).

**TABLE 2:** Gene Ontology Enrichment – Biological Process (Most significant results). Gene ontology biological process enrichment analysis performed on the set of 125 shrew *(Cryptotis parva)* candidate genes for emesis. Term corresponds to the gene ontology term, count indicates how many genes within the 125 candidate gene set contained the biological process annotation, % is the percent of genes containing the annotation within the emesis candidate gene set, *p*-value is the uncorrected *p*-value, Benjamini corresponds to the false discovery rate associated with type I errors in null hypothesis in cases of multiple testing. Complete set of gene ontology biological process enrichment data in Supplementary Table S6.

Term	Count	%	*p*-value	Benjamini
intracellular signal transduction	34	27.9	2.70E-26	2.50E-23
release of sequestered calcium ion into cytosol	16	13.1	1.10E-22	5.00E-20
platelet activation	20	16.4	3.70E-21	1.10E-18
activation of adenylate cyclase activity	15	12.3	6.40E-21	1.50E-18
adenylate cyclase-inhibiting G-protein coupled receptor signaling pathway	15	12.3	9.00E-20	1.70E-17
phosphatidylinositol biosynthetic process	15	12.3	2.50E-18	3.90E-16
inositol phosphate metabolic process	14	11.5	5.90E-18	7.80E-16
lipid catabolic process	16	13.1	2.30E-17	2.70E-15
protein phosphorylation	26	21.3	9.50E-16	1.00E-13
phosphatidylinositol 3-kinase signaling	11	9	1.50E-15	1.50E-13
calcium ion transport	14	11.5	5.40E-15	4.60E-13
serotonin receptor signaling pathway	9	7.4	5.80E-15	4.50E-13
phosphatidylinositol-3-phosphate biosynthetic process	12	9.8	3.00E-14	2.10E-12
chemical synaptic transmission	19	15.6	7.20E-14	4.80E-12
cAMP biosynthetic process	9	7.4	1.10E-13	6.70E-12
activation of protein kinase A activity	9	7.4	1.90E-13	1.10E-11
cyclic nucleotide biosynthetic process	8	6.6	5.40E-13	3.00E-11
peptidyl-serine phosphorylation	14	11.5	4.10E-12	2.10E-10
cAMP-mediated signaling	10	8.2	4.20E-12	2.10E-10
phosphatidylinositol-mediated signaling	13	10.7	1.00E-11	4.80E-10
renal water homeostasis	9	7.4	4.30E-11	1.90E-09
phosphatidylinositol phosphorylation	12	9.8	5.40E-11	2.30E-09
adenylate cyclase-activating G-protein coupled receptor signaling pathway	10	8.2	6.00E-11	2.50E-09
calcium-mediated signaling	10	8.2	7.30E-11	2.80E-09
regulation of cardiac conduction	10	8.2	1.80E-10	6.60E-09
cellular response to glucagon stimulus	9	7.4	3.00E-10	1.10E-08
G-protein coupled receptor signaling pathway, coupled to cyclic nucleotide second messenger	9	7.4	9.80E-10	3.40E-08
Fc-epsilon receptor signaling pathway	13	10.7	4.50E-09	1.50E-07
phospholipase C-activating G-protein coupled receptor signaling pathway	9	7.4	1.90E-08	6.20E-07
regulation of phosphatidylinositol 3-kinase activity	5	4.1	8.00E-08	2.50E-06
vasoconstriction	6	4.9	9.10E-08	2.70E-06
calcium ion transmembrane transport	10	8.2	1.60E-07	4.50E-06
cellular response to forskolin	5	4.1	1.60E-07	4.50E-06
regulation of cardiac muscle contraction by regulation of the release of sequestered calcium ion	6	4.9	1.70E-07	4.60E-06
Fc-gamma receptor signaling pathway involved in phagocytosis	10	8.2	2.70E-07	7.30E-06
regulation of behavior	5	4.1	2.80E-07	7.40E-06
Wnt signaling pathway, calcium modulating pathway	7	5.7	2.90E-07	7.20E-06
cellular calcium ion homeostasis	9	7.4	2.90E-07	7.10E-06
T cell receptor signaling pathway	10	8.2	9.90E-07	2.40E-05
response to amphetamine	6	4.9	2.30E-06	5.40E-05

**TABLE 3:** Gene Ontology Enrichment – Molecular Function (Most significant results). Gene ontology molecular function enrichment analysis performed on the set of 125 shrew (*Cryptotis parva*) candidate genes for emesis. Term corresponds to the gene ontology term, count indicates how many genes within the 125 candidate gene set contained the molecular function annotation, % is the percent of genes containing the annotation within the emesis candidate gene set, *p*-value is the uncorrected *p*-value, Benjamini corresponds to the false discovery rate associated with type I errors in null hypothesis in cases of multiple testing. Complete set of gene ontology molecular function enrichment data in Supplementary Table S7.

Term	Count	%	*p*-value	Benjamini
phosphatidylinositol phospholipase C activity	14	11.5	7.90E-22	1.60E-19
kinase activity	21	17.2	2.60E-16	2.30E-14
ATP binding	43	35.2	5.70E-16	3.80E-14
calmodulin binding	19	15.6	8.10E-16	4.00E-14
G-protein coupled serotonin receptor activity	11	9	2.00E-15	8.20E-14
phospholipase C activity	9	7.4	5.20E-15	1.80E-13
1-phosphatidylinositol-3-kinase activity	12	9.8	5.30E-15	1.50E-13
calmodulin-dependent protein kinase activity	10	8.2	1.20E-14	3.20E-13
serotonin binding	8	6.6	7.60E-14	1.70E-12
neurotransmitter receptor activity	10	8.2	7.60E-14	1.60E-12
protein serine/threonine kinase activity	22	18	1.30E-13	2.40E-12
adenylate cyclase activity	9	7.4	3.00E-13	5.10E-12
phosphorus-oxygen lyase activity	8	6.6	4.90E-13	7.80E-12
1-phosphatidylinositol-4-phosphate 3-kinase activity	7	5.7	6.80E-13	9.90E-12
signal transducer activity	17	13.9	7.90E-13	1.10E-11
protein kinase C activity	8	6.6	3.90E-12	5.00E-11
protein kinase activity	17	13.9	3.60E-09	4.30E-08
calcium ion binding	23	18.9	3.60E-09	4.10E-08
phosphatidylinositol 3-kinase activity	5	4.1	1.10E-08	1.20E-07
1-phosphatidylinositol-3-kinase regulator activity	5	4.1	3.30E-08	3.30E-07
phosphatidylinositol-4,5-bisphosphate 3-kinase activity	8	6.6	2.30E-07	2.20E-06
inositol 1,4,5 trisphosphate binding	5	4.1	7.00E-07	6.50E-06
calcium-release channel activity	5	4.1	7.00E-07	6.50E-06
insulin receptor substrate binding	4	3.3	5.10E-05	4.60E-04
titin binding	4	3.3	1.10E-04	9.50E-04
ion channel binding	7	5.7	1.30E-04	1.10E-03
inositol 1,4,5-trisphosphate-sensitive calcium-release channel activity	3	2.5	1.40E-04	1.10E-03
N-terminal myristoylation domain binding	3	2.5	1.40E-04	1.10E-03
calcium-independent protein kinase C activity	3	2.5	1.40E-04	1.10E-03
Ras guanyl-nucleotide exchange factor activity	7	5.7	1.50E-04	1.10E-03
drug binding	6	4.9	1.80E-04	1.30E-03
phospholipase binding	4	3.3	2.50E-04	1.70E-03
calcium-dependent protein kinase C activity	3	2.5	2.80E-04	1.90E-03
ryanodine-sensitive calcium-release channel activity	3	2.5	2.80E-04	1.90E-03
calcium-induced calcium release activity	3	2.5	2.80E-04	1.90E-03
phosphatidylinositol 3-kinase regulator activity	3	2.5	2.80E-04	1.90E-03
phosphatidylinositol binding	6	4.9	3.10E-04	2.00E-03
protein phosphatase activator activity	3	2.5	7.00E-04	4.50E-03
nitric-oxide synthase regulator activity	3	2.5	1.30E-03	8.00E-03

**TABLE 4:** Gene Ontology Enrichment – Cellular Compartment. Gene ontology cellular compartment enrichment analysis performed on the set of 125 shrew (*Cryptotis parva*) candidate genes for emesis. Term corresponds to the gene ontology term, count indicates how many genes within the 125 candidate gene set contained the cellular component annotation, % is the percent of genes containing the annotation within the emesis candidate gene set, *p*-value is the uncorrected *p*-value, Benjamini corresponds to the false discovery rate associated with type I errors in null hypothesis in cases of multiple testing.

Term	Count	%	*p*-value	Benjamini
phosphatidylinositol 3-kinase complex	13	10.7	3.80E-25	6.10E-23
plasma membrane	79	64.8	5.70E-23	4.60E-21
Intracellular	45	36.9	3.80E-20	2.00E-18
sarcoplasmic reticulum membrane	10	8.2	8.50E-13	3.40E-11
sarcoplasmic reticulum	9	7.4	4.70E-11	1.50E-09
Cytosol	54	44.3	6.20E-11	1.70E-09
calcium channel complex	8	6.6	2.10E-10	4.80E-09
Dendrite	17	13.9	7.40E-10	1.50E-08
platelet dense tubular network membrane	6	4.9	1.50E-09	2.60E-08
integral component of plasma membrane	28	23	4.60E-07	7.30E-06
postsynaptic density	9	7.4	3.20E-05	4.60E-04
Membrane	32	26.2	3.20E-05	4.30E-04
phosphatidylinositol 3-kinase complex, class IB	3	2.5	1.30E-04	1.60E-03
smooth endoplasmic reticulum	4	3.3	6.60E-04	7.60E-03
protein complex	9	7.4	6.20E-03	6.50E-02
growth cone	5	4.1	7.50E-03	7.30E-02
Z disc	5	4.1	7.90E-03	7.30E-02
secretory granule membrane	3	2.5	8.50E-03	7.30E-02
endocytic vesicle membrane	4	3.3	9.60E-03	7.90E-02
phosphatidylinositol 3-kinase complex, class IA	2	1.6	1.30E-02	1.00E-01
voltage-gated calcium channel complex	3	2.5	1.60E-02	1.10E-01
cytoplasm	47	38.5	1.60E-02	1.10E-01
axon	6	4.9	1.60E-02	1.10E-01
sarcomere	3	2.5	2.80E-02	1.70E-01
cell-cell junction	5	4.1	2.80E-02	1.70E-01
phosphatidylinositol 3-kinase complex, class III	2	1.6	3.30E-02	1.90E-01
perikaryon	4	3.3	3.40E-02	1.80E-01
spindle microtubule	3	2.5	3.50E-02	1.80E-01
L-type voltage-gated calcium channel complex	2	1.6	3.90E-02	2.00E-01
late endosome	4	3.3	4.80E-02	2.30E-01
vesicle	4	3.3	5.40E-02	2.50E-01
junctional sarcoplasmic reticulum membrane	2	1.6	6.40E-02	2.80E-01
caveola	3	2.5	6.90E-02	3.00E-01
cilium	4	3.3	7.90E-02	3.20E-01
cell projection	3	2.5	8.90E-02	3.50E-01

Among the enriched biological processes, one of the most significant results is the association of 16 of the candidate emesis genes with “release of sequestered calcium ion into cytosol” (*p*-value = 1.10E-22) and 14 of the genes annotated with “calcium ion transport” (*p*-value = 5.40E-15). Other noteworthy biological processes identified in the analysis include “serotonin receptor signaling pathway” (*p*-value = 5.80E-15); “G-protein coupled receptor signaling pathway, coupled to cyclic nucleotide second messenger” (*p*-value = 6.00E-11); and “chemical synaptic transmission” (*p*-value = 7.20E-14). Together these results underscore the significant roles mediators of emesis play in calcium signaling and neurotransmitter transduction.

Within the category of molecular function (Table 3), our analysis identified a number of proteins implicated in calcium homeostasis and signaling including 19 genes implicated in “calmodulin binding” (*p*-value = 8.10E-16); 10 genes annotated as having “calmodulin-dependent protein kinase activity” (*p*-value = 1.20E-14); and 23 genes exhibiting “calcium ion binding” (*p*-value = 3.60E-09). Supplementally, proteins molecular functions related to neurotransmitter functions were also identified such as a set of eight genes that bind serotonin (*p*-value = 7.60E-14) and two genes classified as having “tachykinin receptor activity” (*p*-value = 2.10E-02). These important functions are critical to the regulation and pharmacological intervention of emesis pathways.

The cellular compartment category of gene ontology (Table 4) associates specific locations inside and outside of the cell with genes. In our analysis we identified the “phosphatidylinositol 3-kinase complex” (*p*-value = 3.80E-25) as a critical location where 13 genes in the emesis candidate gene set are localized. Supplementally, “sarcoplasmic reticulum membrane” and “sarcoplasmic reticulum” are highly significant with *p*-values of 8.50E-13 and 4.70E-11, respectively. Interestingly, 17 of our genes are associated with neuronal dendrites (*p*-value = 7.40E-10) and nine genes are annotated with the term “postsynaptic density” (*p*-value = 3.20E-05). Together, the biological process, molecular function and cellular compartment provide considerable insight into the biochemistry and neurobiology that these genes mediate.

Although gene ontology enrichment provides clues as to the function and location of specific molecules in our data set, such an analysis does not provide clues as to additional molecular mediators that may also be involved with the genes identified in our candidate gene set. The identification of enriched interacting proteins (Table 5) overcomes this limitation by utilizing information about protein-protein interactions and the macromolecular associations of molecules in specific complexes within the cell. This analysis looks at the set of input genes and seeks to identify clusters of genes that interact with a common partner. Such common partners may include novel pharmacological targets, or other mediators of the cellular biology under investigation.

**TABLE 5:** Enriched Interacting Proteins – Shrew Emesis Genes. Enrichment interacting proteins analysis performed on the set of 125 shrew (*Cryptotis parva*) candidate genes for emesis. Term corresponds to the specific enriched interacting protein, count indicates how many genes within the 125 candidate gene set contained the interacting protein annotation, % is the percent of genes containing the annotation within the emesis candidate gene set, *p*-value is the uncorrected *p*-value, Benjamini corresponds to the false discovery rate associated with type I errors in null hypothesis in cases of multiple testing.

Term	Count	%	*p*-value	Benjamini
calcium/calmodulin dependent protein kinase II delta(CAMK2D)	7	5.7	3.50E-11	2.60E-09
calmodulin 1(CALM1)	7	5.7	8.20E-09	3.00E-07
calmodulin 3(CALM3)	7	5.7	8.20E-09	3.00E-07
calmodulin 2(CALM2)	7	5.7	1.40E-08	3.40E-07
calcium/calmodulin dependent protein kinase II beta(CAMK2B)	4	3.3	1.00E-05	1.90E-04
calcium/calmodulin dependent protein kinase II alpha(CAMK2A)	4	3.3	1.00E-05	1.90E-04
calcium/calmodulin dependent protein kinase II gamma(CAMK2G)	4	3.3	2.60E-05	3.70E-04
myelin basic protein(MBP)	3	2.5	5.80E-04	7.10E-03
troponin I3, cardiac type(TNNI3)	3	2.5	5.80E-04	7.10E-03
calcium/calmodulin dependent protein kinase I(CAMK1)	3	2.5	5.80E-04	7.10E-03
calcium/calmodulin dependent protein kinase IV(CAMK4)	3	2.5	5.80E-04	7.10E-03
potassium calcium-activated channel subfamily N member 2(KCNN2)	3	2.5	1.20E-03	1.20E-02
EPH receptor A3(EPHA3)	3	2.5	1.90E-03	1.70E-02
stromal interaction molecule 1(STIM1)	3	2.5	1.90E-03	1.70E-02
sodium voltage-gated channel alpha subunit 5(SCN5A)	3	2.5	2.80E-03	2.30E-02
SWI/SNF related, matrix associated, actin dependent regulator of chromatin, subfamily b, member 1(SMARCB1)	3	2.5	3.90E-03	2.80E-02
calcium voltage-gated channel subunit alpha1 C(CACNA1C)	3	2.5	5.20E-03	3.40E-02
beclin 1(BECN1)	3	2.5	5.20E-03	3.40E-02
ORAI calcium release-activated calcium modulator 1(ORAI1)	2	1.6	4.20E-02	2.30E-01
biliverdin reductase A(BLVRA)	2	1.6	4.20E-02	2.30E-01

We identified a strong calcium theme among our candidate genes which includes interacting molecules calmodulin1, calmodulin 3 (each with *p*-value = 8.20E-09), and calmodulin 2 (*p*-value = 1.40E-08). The most significant result in our analysis is calcium/calmodulin dependent protein kinase II delta (CAMK2D) for which 7 of our candidate emesis genes interact (*p*-value = 3.50E-11). Other interacting proteins in the analysis include members of the calcium/calmodulin dependent protein kinase II family, as well as calcium/calmodulin dependent protein kinase I (CAMK1) calcium/calmodulin dependent protein kinase IV (CAMK4). One surprising, and yet very relevant detected enriched interacting protein we detected is stromal interaction molecule 1 (STIM1) (*p*-value = 1.90E-03) that transitions from an inactive to an active calcium shuttle under certain conditions to facilitate movement of extracellular calcium. Taken together, these results implicate calcium as a key element in the interactions of the candidate gene set.

To better understand the tissue expression associated with the candidate emesis genes we investigated the enriched UniProt Tissue Expression Database (Table 6) which classifies genes by tissue of expression. The top result for our analysis is “brain”, for which 78 genes were associated (*p*-value = 1.70E-06) and “brainstem” (*p*-value = 2.90E-03). Our analysis also identified six genes expressed in “small intestine” (*p*-value = 7.00E-02).

**TABLE 6:** Enriched UniProt Tissue Expression – Shrew Emesis Genes. Enrichment UniProt tissue expression analysis performed on the set of 125 shrew (*Cryptotis parva*) candidate genes for emesis. Term corresponds to the specific enriched interacting protein, count indicates how many genes within the 125 candidate gene set contained the tissue expression annotation, % is the percent of genes containing the annotation within the emesis candidate gene set, *p*-value is the uncorrected *p*-value, Benjamini corresponds to the false discovery rate associated with type I errors in null hypothesis in cases of multiple testing.

Term	Count	%	*p*-value	Benjamini
Brain	78	63.9	1.70E-06	1.80E-04
Myometrium	3	2.5	1.40E-03	6.70E-02
Brainstem	3	2.5	2.90E-03	9.30E-02
Substantia nigra	4	3.3	5.20E-03	1.20E-01
Hippocampus	9	7.4	9.00E-03	1.70E-01
Corpus striatum	2	1.6	3.10E-02	4.10E-01
Osteosarcoma	3	2.5	3.20E-02	3.80E-01
Primary B-Cells	3	2.5	3.80E-02	3.90E-01
Blood	10	8.2	4.90E-02	4.30E-01
Lymphoma	3	2.5	5.60E-02	4.40E-01
Spleen	11	9	6.10E-02	4.40E-01
Myeloid	2	1.6	6.70E-02	4.40E-01
Lung	25	20.5	6.90E-02	4.30E-01
Small intestine	6	4.9	7.00E-02	4.10E-01
Platelet	8	6.6	7.10E-02	3.90E-01

### 3.7 Identification of conserved one-to-one orthologs of ferret, dog, mouse, and human

We identified a core set of 6,952 one-to-one protein-coding orthologs across all five species, shrew, ferret, dog, mouse, and human. This set of genes provides a common framework for investigating the collective molecular biology across these diverse mammalian species. This set of genes is extremely valuable because it provides a common context for which shared biology can be investigated to uncover both the conserved aspects of biology as well as the differences between species. We performed a large-scale sequence comparison analysis to gain a better understanding of the relationship between the different species.

Our analysis investigated both the nucleotide level and amino acid level identity between the set of 6,952 protein coding conserved orthologs across shrew, dog, ferret, mouse, and human. One question we had was which of the four species were most identical to the shrew sequences. The average nucleotide identity across these 6,999 nucleotide genes and 6,952 protein-coding genes were compared and tested for statistical significance across all “pairs” of datasets, such as shrew-mouse vs shrew-human and shrew-ferret vs shrew-human. The only significant comparisons were those including shrew-mouse (average nucleotide identity 85.937 and average amino acid identity 89.962).

For example, one-tailed test of shrew-human nucleotide identity *versus* shrew-mouse nucleotide identity indicated *p* < 0.00001. In contrast, the average nucleotide identity between the shrew-human *versus* shrew-ferret was so similar that the resulting *p*-value was 0.44038. The analysis for the average protein identity followed this same pattern with all pairs including shrew-mouse exhibiting statistical significance while none of the other comparisons rose to a level of significance given our alpha. The results indicate that the shrew is a good match to human sequences, and although it is not statistically significant, the shrew-human average amino acid identity and the shrew-ferret amino acid identity are both higher than 91% but lower than 92% identical based on our analysis of this data set.

### 3.8 Heat map visualization of candidate emesis gene set identity across species

**FIGURE 5:**
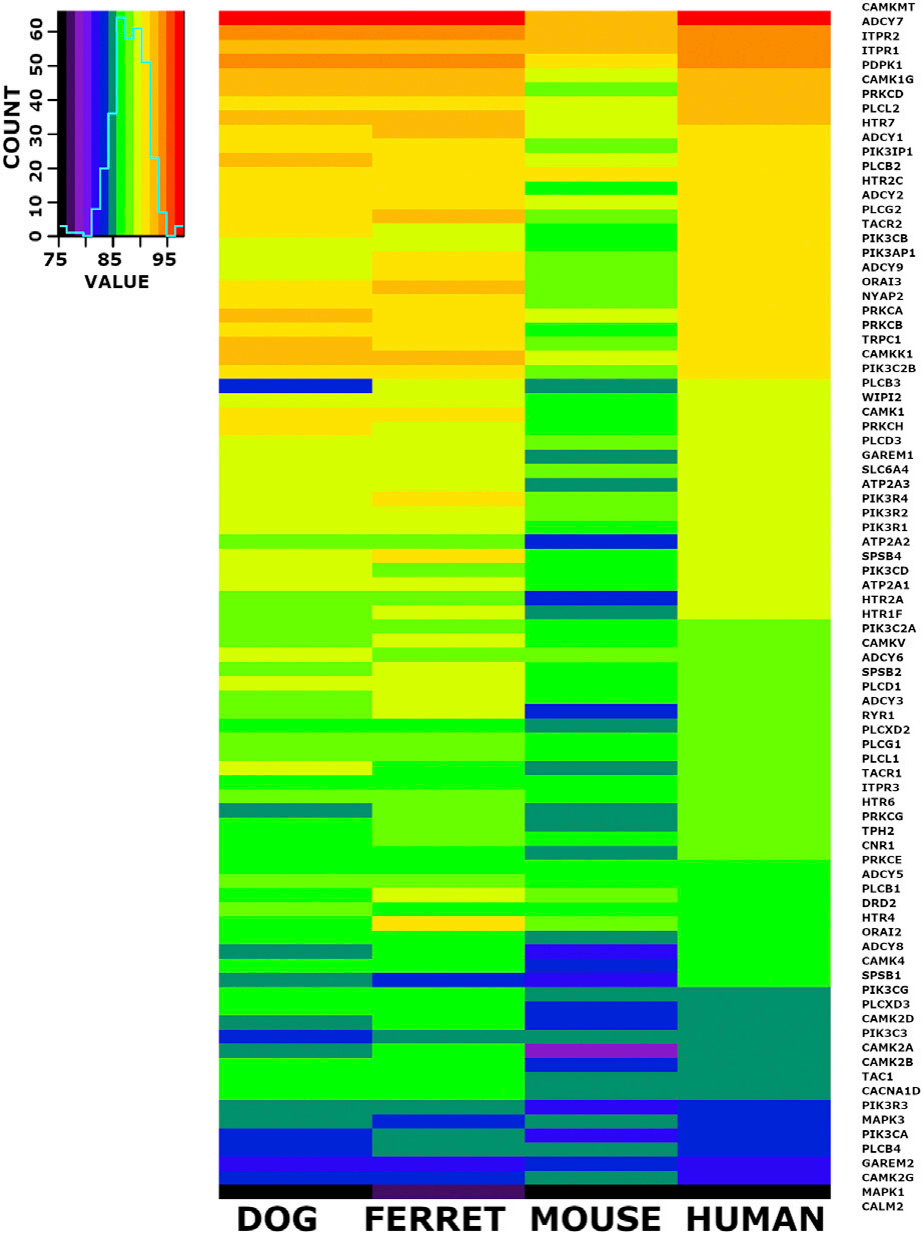
Heat Map Illustrating Identity Between Shrew cDNA and Orthologs of Emesis Candidate Genes. A set of 84 cDNAs from the emesis candidate gene set was compared at the nucleotide level between shrew and dog, ferret, mouse, and human. Results are represented in heat map format. The color key and histogram in the upper left corner provides a mapping between heat map colors and the count of cDNAs in each color category. The data is ordered by the nucleotide identity relationship between shrew cDNAs and corresponding orthologous human cDNAs in the last column.

**FIGURE 6:**
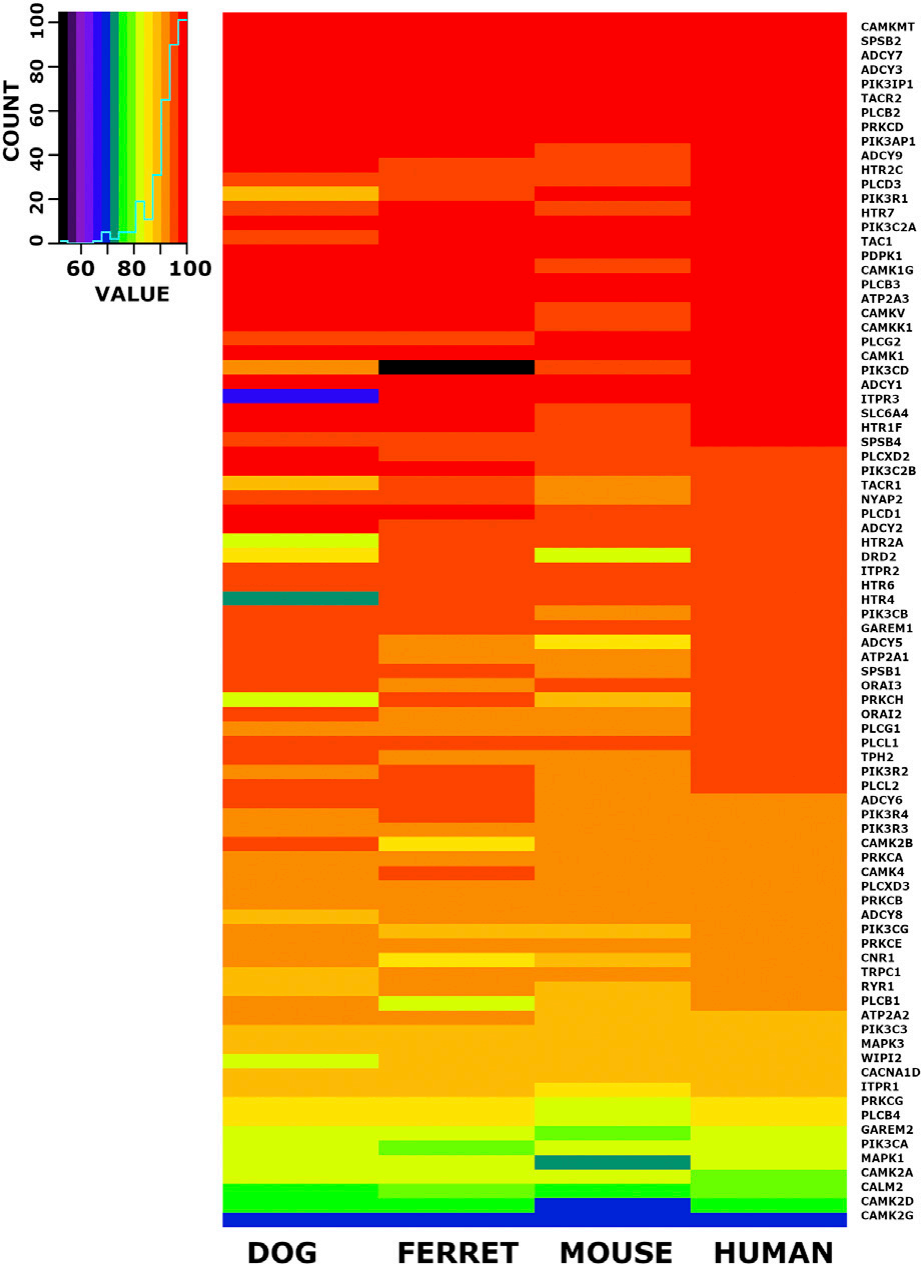
Heat Map Illustrating Identity Between Shrew Protein and Orthologs of Emesis Candidate Genes. A set of 84 cDNAs from the emesis candidate gene set was compared at the inferred amino acid level between shrew and dog, ferret, mouse and human. Results are represented in heat map format. The color key and histogram in the upper left corner provides a mapping between heat map colors and the count of cDNAs in each color category. The data is ordered by the nucleotide identity relationship between shrew cDNAs and corresponding orthologous human cDNAs in the last column.

When we checked the 6,999 conserved cDNA orthologs and the conserved 6,952 protein coding orthologs for members of the emesis candidate genes, we identified 84 candidate genes. The nucleotide percent identity for each of these 84 emesis candidates between the shrew and the dog, ferret, mouse, and human versions of these genes are shown in heatmap form (Figure 5). The data is ordered by the identity relationship between shrew and human in the last column. The same data is displayed for the amino acid identity in translated proteins (Figure 6).

### 3.9 Phenotype enrichment of most conserved protein coding orthologs

We investigated the phenotype enrichment for genes belonging to the top 10% most conserved 6,952 orthologs (Table 7, Supplemental File 8). This analysis sought to identify disproportionate phenotypes that were enriched in the top 10% *versus* the rest of the genome. We identified signals from developmental, embryonic, neonatal and lethality related phenotypes. These phenotypes included mortality/aging (p = 7E-22); prenatal lethality (*p* = 1.1E-12); preweaning lethality (*p* = 1.6E-27); lethality during fetal growth through weaning (*p* = 1.0E-06), perinatal lethality (*p* = 1.2E-05) and embryo phenotype (*p* = 6.1E-06).

**TABLE 7:** Phenotype Enrichment of 692 Most Conserved Orthologs (Subset of Most significant results). Phenotype enrichment for the top 10% most conserved genes from among the core set of 6952 one-to-one protein-coding orthologs across all five species, shrew, ferret, dog, mouse, human and dog. Subset of all phenotype enrichment data (Supplementary Table S8) provides a representative set of enriched phenotypes implicated in development, embryonic/neonatal lethality as well as nervous system morphology, neurotransmitter related phenotypes. Fisher’s exact test corresponds to uncorrected p-value, FDR corresponds to False Discovery Rate, and Bonferroni refers to Bonferroni correction for multiple testing. Full enriched phenotypes are available in Supplementary Table S8.

Enriched Phenotype	Fisher’sTest	FDR	Bonferroni
■ mortality/aging	2.493E-23	6.98E-22	6.9804E-22
└ abnormal survival	2.268E-29	1.588E-27	1.5876E-27
├ preweaning lethality	5.123E-29	1.609E-26	1.60862E-26
│├ prenatal lethality	1.843E-15	1.1E-12	1.10027E-12
├ perinatal lethality	3.947E-08	0.000004131	1.23936E-05
│├ neonatal lethality	2.874E-07	0.00004289	0.000171578
││├ neonatal lethality, complete penetrance	0.000001889	0.0002599	0.001354413
■ embryo phenotype	2.186E-07	0.00000306	6.1208E-06
├ abnormal embryo development	0.000003449	0.00008048	0.00024143
│├ abnormal developmental patterning	0.00007631	0.002	0.02396134
││├ abnormal gastrulation	0.0005583	0.019	0.3333051
│││├ failure to gastrulate	0.00005456	0.003	0.03911952
├ abnormal embryo morphology	0.000008526	0.0001492	0.00059682
│├ abnormal embryo size	0.0004737	0.011	0.1487418
■ growth/size/body region phenotype	0.000001478	0.00001379	0.000041384
├ abnormal prenatal growth/weight/body size	0.000001531	0.00005359	0.00010717
│├ abnormal embryonic growth/weight/body size	0.00000816	0.0006406	0.00256224
││├ abnormal embryo size	0.0004737	0.017	0.2827989
│├ abnormal prenatal body size	0.00007949	0.002	0.02495986
│├ abnormal postnatal growth	0.00004378	0.002	0.01374692
││└ postnatal growth retardation	0.00008227	0.004	0.04911519
■ nervous system phenotype	0.004	0.028	0.112
├ abnormal nervous system morphology	0.0002129	0.003	0.014903
│├ abnormal brain morphology	0.00003144	0.002	0.00987216
││├ abnormal hindbrain morphology	0.000009265	0.0006914	0.005531205
│││├ abnormal metencephalon morphology	0.00003414	0.002	0.02447838
││││├ abnormal cerebellum morphology	0.0005448	0.034	0.3437688
│││││├ abnormal cerebellar cortex morphology	0.0004864	0.024	0.2120704
│││││││├ abnormal cerebellar molecular layer	0.0001185	0.003	0.0235815
││││││││├ abnormal cerebellar lobule formation	0.00002125	0.00051	0.00255
││││││ ├ abnormal hippocampus neuron morphology	0.00002004	0.0006041	0.00398796
││││││ │└ abnormal hippocampus pyramidal cell morphology	0.000002175	0.0001305	0.000261
││││││ │ ├ ectopic hippocampus pyramidal cells	0.000001957	0.0000522	0.000093936
││││││ │ ├ decreased hippocampus pyramidal cell number	0.0001548	0.002	0.0074304
│├ abnormal neuron morphology	0.0003297	0.009	0.1035258
││├ ectopic neuron	0.000005499	0.000469	0.003282903
│││├ ectopic hippocampus pyramidal cells	0.000001957	0.0002599	0.001403169
│││└ ectopic Purkinje cell	0.000458	0.019	0.328386
││├ abnormal hippocampus neuron morphology	0.00002004	0.001	0.01196388
│││└ abnormal hippocampus pyramidal cell morphology	0.000002175	0.0002599	0.001559475
│││ ├ ectopic hippocampus pyramidal cells	0.000001957	0.000247	0.001234867
│││ ├ decreased hippocampus pyramidal cell number	0.0001548	0.012	0.0976788
├ abnormal synaptic transmission	0.000213	0.006	0.066882
│├ abnormal CNS synaptic transmission	0.000002046	0.0002036	0.001221462
││├ abnormal glutamate-mediated receptor currents	0.000003345	0.0003124	0.002398365
│││└ abnormal AMPA-mediated synaptic currents	4.087E-07	0.00006447	0.00025789
│││ ├ reduced AMPA-mediated synaptic currents	0.0006632	0.029	0.2891552
││├ abnormal long term potentiation	0.000266	0.014	0.190722
│││├ enhanced long term potentiation	0.011	0.198	1
│││└ reduced long term potentiation	0.034	0.306	1
││├ abnormal excitatory postsynaptic potential	0.0006	0.024	0.4302
││├ abnormal synaptic depression	0.002	0.055	1
│││└ abnormal long term depression	0.0006099	0.035	0.3848469
│││ ├ absent long term depression	0.001	0.034	0.436
├ abnormal nervous system electrophysiology	0.001	0.02	0.314

We also identified strong signals from phenotypes associated with brain formation and morphology. Phenotypes in this category included abnormal brain morphology (*p* = 0.00987); abnormal hindbrain morphology (*p* = 0.00553); abnormal cerebellar lobule formation (*p* = 0.00255); abnormal limbic system morphology (*p* = 0.00541); abnormal hippocampus neuron morphology (*p* = 0.00874); and decreased hippocampus pyramidal cell number (*p* = 0.00279).

Lastly a small signal from phenotypes implicated in neurotransmission was also identified in the data. Phenotypes in this category included abnormal CNS synaptic transmission (*p* = 0.00122); abnormal glutamate-mediated receptor currents (*p* = 0.0024); and abnormal AMPA-mediated synaptic currents (*p* = 0.00025789). Other phenotypes include ataxia (*p* = 0.01782) and abnormal motor coordination/balance (*p* = 0.05543 which lies just shy of significance).

### 3.10 KEGG pathway enrichment of most conserved protein coding orthologs

Similar to the phenotype enrichment, we analyzed the top 10% of the 6952-protein coding conserved orthologs to identify specific cellular and biochemical pathways that were enriched with members among the genes in that dataset (Table 8). Notable pathways could be organized into roughly general categories corresponding to neurotransmitter signaling, neuronal pathways and memory related pathways, generic signaling pathways and addiction related pathways.

**TABLE 8:** KEGG Pathway Enrichment of 692 Most Conserved Orthologs. Pathway enrichment for the top 10% most conserved genes from among the core set of 6952 one-to-one protein-coding orthologs across all five species, shrew, ferret, dog, mouse, human and dog. Set of all pathway enrichment data provides a representation of the biochemical and signaling networks formed by the most conserved orthologs. Among the enriched pathways are neurotransmitter associated pathways (including dopamine synapse, serotonin synapse, oxytocin, adrenergic signaling, endocannabinoid signaling and GABA/Glutaminergic signaling), memory related pathways (such as long term potentiation, long term depression, and axon guidance) as well as neurobiology pathways associated with alcohol and amphetamine addiction. Count corresponds to the number of genes within the top 10% most conserved that were associated with the pathway, *p*-value corresponds to uncorrected *p*-value, Bonferroni refers to Bonferroni correction for multiple testing, and Benjamini corresponds to False Discovery Rate.

KEGG pathway	Count	*p*-value	Bonferroni	Benjamini
hsa03040:Spliceosome	33	1.93E-14	3.88E-12	3.88E-12
hsa04728:Dopaminergic synapse	30	1.78E-12	3.58E-10	1.79E-10
hsa04720:Long-term potentiation	18	1.01E-08	2.03E-06	6.77E-07
hsa04713:Circadian entrainment	21	2.14E-08	4.30E-06	1.08E-06
hsa04120:Ubiquitin mediated proteolysis	25	3.70E-08	7.43E-06	1.49E-06
hsa04261:Adrenergic signaling in cardiomyocytes	24	1.86E-07	3.73E-05	6.22E-06
hsa04921:Oxytocin signaling pathway	25	2.21E-07	4.43E-05	6.33E-06
hsa03015:mRNA surveillance pathway	19	3.00E-07	6.03E-05	7.54E-06
hsa04114:Oocyte meiosis	21	3.26E-07	6.55E-05	7.28E-06
hsa04725:Cholinergic synapse	21	3.26E-07	6.55E-05	7.28E-06
hsa05216:Thyroid cancer	11	6.06E-07	1.22E-04	1.22E-05
hsa04390:Hippo signaling pathway	22	1.27E-05	0.002542	2.31E-04
hsa04730:Long-term depression	13	2.62E-05	0.005247	4.38E-04
hsa04024:cAMP signaling pathway	25	3.24E-05	0.006495	5.01E-04
hsa05210:Colorectal cancer	13	3.70E-05	0.007405	5.31E-04
hsa04310:Wnt signaling pathway	20	3.80E-05	0.007605	5.09E-04
hsa04722:Neurotrophin signaling pathway	18	6.75E-05	0.013477	8.48E-04
hsa05031:Amphetamine addiction	13	7.06E-05	0.014091	8.34E-04
hsa05211:Renal cell carcinoma	13	7.06E-05	0.014091	8.34E-04
hsa04012:ErbB signaling pathway	15	7.29E-05	0.014555	8.14E-04
hsa05200:Pathways in cancer	38	8.20E-05	0.016339	8.67E-04
hsa04916:Melanogenesis	16	9.32E-05	0.018569	9.37E-04
hsa04723:Retrograde endocannabinoid signaling	16	1.05E-04	0.020833	0.001002
hsa04724:Glutamatergic synapse	17	1.23E-04	0.024519	0.001128
hsa04360:Axon guidance	18	1.39E-04	0.027464	0.00121
hsa05213:Endometrial cancer	11	1.75E-04	0.034511	0.001462
hsa03050:Proteasome	10	2.26E-04	0.044366	0.001814
hsa04010:MAPK signaling pathway	27	2.51E-04	0.04928	0.001942
hsa03010:Ribosome	18	3.20E-04	0.062394	0.002383
hsa05034:Alcoholism	21	3.83E-04	0.074084	0.002745
hsa04520:Adherens junction	12	6.03E-04	0.114137	0.00417
hsa04071:Sphingolipid signaling pathway	16	7.14E-04	0.133822	0.004777
hsa04727:GABAergic synapse	13	8.16E-04	0.151387	0.005281
hsa04152:AMPK signaling pathway	16	9.27E-04	0.170052	0.005808
hsa04726:Serotonergic synapse	15	9.77E-04	0.178303	0.005933
hsa04550:Signaling pathways regulating pluripotency of stem cells	17	0.001283	0.227405	0.00756
hsa05221:Acute myeloid leukemia	10	0.001436	0.250945	0.008222
hsa04144:Endocytosis	24	0.00156	0.269305	0.008678
hsa05142:Chagas disease (American trypanosomiasis)	14	0.001597	0.274757	0.008645
hsa04150:mTOR signaling pathway	10	0.001853	0.311139	0.00976
hsa04068:FoxO signaling pathway	16	0.002218	0.360017	0.011379
hsa04110:Cell cycle	15	0.002844	0.435828	0.014208
hsa04910:Insulin signaling pathway	16	0.00296	0.448862	0.014426
hsa04915:Estrogen signaling pathway	13	0.003103	0.464579	0.014764
hsa04922:Glucagon signaling pathway	13	0.003103	0.464579	0.014764
hsa04660:T cell receptor signaling pathway	13	0.003375	0.493172	0.01568
hsa04014:Ras signaling pathway	22	0.003409	0.496644	0.01548
hsa04919:Thyroid hormone signaling pathway	14	0.003931	0.546918	0.017439
hsa05212:Pancreatic cancer	10	0.004117	0.563651	0.017867
hsa05214:Glioma	10	0.004117	0.563651	0.017867
hsa04022:cGMP-PKG signaling pathway	17	0.004435	0.590754	0.01883
hsa04912:GnRH signaling pathway	12	0.004655	0.608542	0.019349
hsa05160:Hepatitis C	15	0.00538	0.661851	0.021885
hsa04810:Regulation of actin cytoskeleton	20	0.006965	0.754612	0.027707
hsa05202:Transcriptional misregulation in cancer	17	0.007546	0.781819	0.02941
hsa04666:Fc gamma R-mediated phagocytosis	11	0.00761	0.784626	0.029095
hsa04350:TGF-beta signaling pathway	11	0.00761	0.784626	0.029095
hsa04141:Protein processing in endoplasmic reticulum	17	0.008432	0.817686	0.031603
hsa05205:Proteoglycans in cancer	19	0.008973	0.836637	0.032995
hsa04370:VEGF signaling pathway	9	0.009282	0.846547	0.033505
hsa04062:Chemokine signaling pathway	18	0.009458	0.851923	0.033532
hsa03013:RNA transport	17	0.009916	0.86507	0.03453
hsa05215:Prostate cancer	11	0.010474	0.879535	0.035832
hsa04721:Synaptic vesicle cycle	9	0.01122	0.896469	0.037709
hsa05130:Pathogenic Escherichia coli infection	8	0.01148	0.901802	0.037941
hsa04924:Renin secretion	9	0.012293	0.916782	0.039939
hsa05032:Morphine addiction	11	0.013112	0.929555	0.041887
hsa05100:Bacterial invasion of epithelial cells	10	0.013485	0.934706	0.04239

Among the neurotransmitter signaling pathways are Dopaminergic synapse (*p* = 1.78E-12); Cholinergic synapse (*p* = 3.26E-07); Adrenergic signaling in cardiomyocytes (*p* = 1.86E-07); Oxytocin signaling pathway (*p* = 2.21E-07); Glutamatergic synapse (*p* = 1.23E-04); and GABAergic synapse (*p* = 8.16E-04). Within the neuronal and memory related pathways we identified Long-term potentiation (*p* = 1.01E-08); Circadian entrainment (*p* = 2.14E-08; Long-term depression (*p* = 2.62E-05); Axon guidance (*p* = 1.39E-04); Neurotrophin signaling pathway (*p* = 6.75E-05); and Synaptic vesicle cycle (*p* = 0.0112).

Among the generic pathways we detected PI3K-Akt signaling pathway (*p* = 0.0315); cAMP signaling pathway (*p* = 3.24E-05); Sphingolipid signaling pathway (*p* = 7.14E-04); HIF-1 signaling pathway (*p* = 0.0185); and Gap junction (*p* = 0.0276).

Finally, the addiction related pathways we identified included Cocaine addiction (*p* = 0.090 may indicate a trending pathway); Amphetamine addiction (*p* = 7.06E-05); Alcoholism (*p* = 3.83E-04); and Morphine addiction (*p* = 0.0441). Together, signaling processes are highly enriched for pathways that are important neurobiology signals and processes. Moreover, the phenotype and KEGG pathway analysis provide insight into these most conserved shared orthologs expressed in brainstem and gut.

### 3.11 Emesis pathway members and identification of best hit orthologous transcripts

**FIGURE 7:**
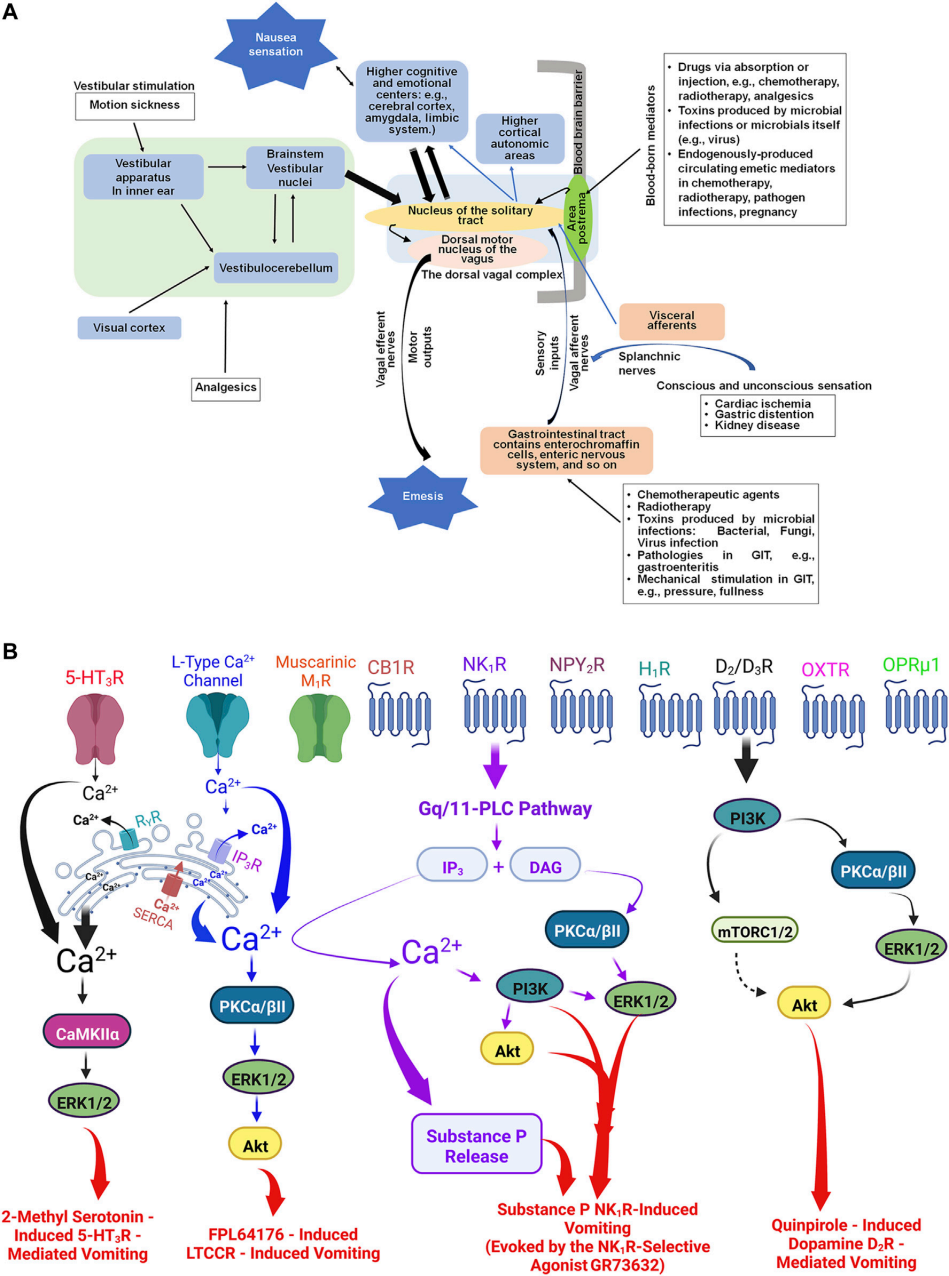
**(A)**: The central and peripheral anatomical sites in the mediation of nausea and vomiting evoked by diverse stimuli. Following exposure to various emetics, nausea and vomiting can be generated *via* bidirectional interactions between brain and the gastrointestinal tract (GIT). Briefly: 1) the brainstem area postrema in the floor of the fourth ventricle lacks blood brain barrier and allows circulating emetogens direct entry from the circulating blood into the cerebrospinal fluid and the brain tissue (Wickham, 2020); 2) systemically administered emetogens can activate corresponding receptors present on peripheral vagal afferents in the gastrointestinal tract, which project sensory emetic signals to the nucleus of the solitary tract (Navari, 2014; Wickham, 2020); and 3) peripheral emetics that enter the lumen of GIT such as cytotoxic chemotherapeutics, or microbials (e.g., bacteria, viruses, fungi), as well as gastrointestinal pathologies, can cause release of local emetic neurotransmitters/hormones, which subsequently act on the corresponding receptors present on vagal afferents and/or directly stimulate the brainstem area postrema *via* circulating blood (Bashashati and McCallum, 2014; McKenzie et al., 2019). Besides the area postrema and the sensory vagal afferents, the nucleus of the solitary tract is also the recipient of: i) direct neural inputs from the splanchnic nerves carrying sensation caused by diseases of visceral organs (e.g., cardiac, kidney); ii) brainstem vestibular nuclei collecting signals from vestibular apparatus in inner ear and/or cerebellum, caused by stimuli related to motion sickness and opioid analgesics (Porreca and Ossipov, 2009; Smith and Laufer, 2014) and iii) the cerebral cortex and limbic system, which accept and process emotional and cognitive stimuli (Zhong et al., 2021). The nucleus of the solitary tract has efferent pathways to the dorsal motor nucleus of the vagus, which further project to the upper gastrointestinal tract to produce the vomiting reflex (Wickham, 2020). In addition, the nucleus of the solitary tract has projections to the mid- and forebrain areas for the perception of nausea (Horn et al., 2014). **(B)** Examples of some of the well-investigated emetic receptors include: Serotonin 5-HT_3_, dopamine D_2_/D_3_, substance P neurokinin NK_1_, L-type Calcium channel, muscarinic M_1_, cannabinoid CB_1_, neuropeptide Y2, histamine H_1_, opioid μ, and oxytocin receptor. Some examples of published intracellular signaling emetic cascades evoked by their corresponding receptor selective agonists in the least shrew include: i) serotonin 5-HT_3_ receptors activated by corresponding selective agonist 2-methyl serotonin (Zhong et al., 2014b); ii) quinpirole-evoked stimulation of dopamine D_2/3_ receptors (Belkacemi et al., 2021); iii) L-type selective calcium agonist FPL64176 (Zhong et al., 2018); and the selective substance P neurokinin NK_1_ receptor selective agonist GR73632 (Zhong et al., 2019). For simplicity, we have not shown the intracellular emetic signals such as following inhibition of phosphodiesterase four enzyme by rolipram-like drugs which elevate intracellular levels of c-AMP signaling in least shrews (Alkam et al., 2014) and ferrets (Robichaud et al., 1999). Abbreviations: PLC, phospholipase C; AP, area postrema; NTS, nucleus tractus solitarius; DMNV, dorsal motor nucleus of the vagus; GPCRs, G protein-coupled receptors; 5-HT_3_R, Serotonin type 3 receptor; NK_1_R, Substance P neurokinin one receptor; D_2/3_R, dopamine D_2_ and D_3_ receptors; LTCCR, L-type Ca^2+^ channel receptor; OXTR, oxytocin receptor, M_1_R, muscarinic M1 receptor; CB_1_R, Cannabinoid CB1 receptor; NPY_2_R, neuropeptide Y receptor type 2; OPRμ1, Opioid receptor μ1; H_1_R, Histamine one receptor; IP_3_R, inositol-1, 4, 5-triphosphate receptor; RyR, ryanodine receptor; DAG, diacylglycerol; PKCα/βII, protein kinase C α/βII; ERK1/2, extracellular signal-regulated protein kinase1/2; Akt, protein kinase B (PKB); CaMKIIα, Ca^2+^/calmodulin-dependent kinase II; mTORC1/2, mammalian targets of rapamycin complexes C1/C2.; PI3K, phosphoinositide 3-kinase; 5-HT, serotonin; SERCA, sarco/endoplasmic reticulum Ca^2+^-ATPase; CINV, chemotherapy-induced nausea and vomiting; Ca^2+^, calcium.

We used the recently published (Zhong et al., 2021) the model of central and peripheral nervous system involved in emesis (Figure 7A) to identify a final set of 39 emesis receptors and signal transduction components (Figure 7B) from the initial set of 16,720 ortholgous gene sequences. These pathway components represent the currently known signal transduction components that mediate the effects of agonists and antagonists of emesis in the well-studied least shrew model. Among these pathway members include dopamine D_2_/D_3_-, opioid μ, neurokinin NK_1-_ and serotonin 5-HT_3_-receptors as well as receptors for oxytocin and neuropeptide Y. Intracellular transduction machinery we identified includes members of well-known gene families such as L-type calcium channels, Protein Kinase C, Mitogen Activated Kinase C, and Phospholipase C. We identified the top match for each of our pathway members across human, dog, mouse, ferret, and shrew. These results provide valuable information about the least shrew genes mediating emesis and their corresponding orthologs in other species of interest. We identified the top scoring hit for each least shrew transcript (Supplemental File 10) we identified across human, dog, ferret, mouse, shrew (*Sorex araneus*)*.*

## 4 Discussion

In this paper we have described the sequencing and analysis of shrew brainstem and gut transcriptomes. We identified a set of 16,720 assembled transcripts that exhibit orthology to human, mouse, dog and/or ferret sequences. This study was carried out to expand knowledge and understanding of emesis pathways. Interestingly, several mammalian species vomit, including humans, dogs, ferrets, and shrews. Other species such as mice and rats, do not vomit. In addition, among different mammalian species, the neurokinin NK_1_ receptor is classified as human-like (e.g., ferret, least shrew)- or rodent-like (e.g., mice, rats) (Darmani et al., 2013). The initial selective antagonists for NK_1_ receptors were developed against rodent-like NK_1_ receptors which had little affinity for the human-like receptors (Rojas and Slusher, 2015). Furthermore, the ferret and shrew have been model organisms for emetic research, therefore understanding the relationship between the genes amongst these species with differing phenotypes helps provide a context for understanding the findings that have been discovered so far. The inclusion of mouse, which does not vomit, helps provide insight into the role homology may (or may not) play in phenotypic differences observed between these two species.

Even though interspecies genomic variation may contribute to differences in the emetic phenotypes, other factors including post-translational modifications, gene expression, localization and phosphorylation status may alter emesis phenotypes. The application of this research includes both human (Hesketh, 2008) and veterinary patients, including dogs and cats (Kobrinsky et al., 1988). These species are routinely diagnosed with many of the same types of cancers and treated with similar chemotherapeutic agents. Subsequently, the inclusion of dog, ferret, human and mouse provides the necessary comparative genomics data for fully leveraging the least shrew in future emesis research.

During our analysis we characterized the length distribution of these sequences and demonstrated that the vast majority of these new shrew cDNA sequences have length of at least 1,000 bp. To assess whether our sequences showed any identity to previously deposited shrew sequences we compared three previously published shrew sequences (Brain Derived Neurotrophic Factor Transcript Sequence [GenBank: LC124902.1] (Sato et al., 2016), Neurokinin Receptor 1 Transcript Sequence [GenBank: JQ715623.1] (Darmani et al., 2013) and Tachykinin precursor one Transcript Sequence [GenBank: FJ696706.1] (Dey et al., 2010). In all three cases, our newly assembled transcript sequence was both 100% identical to the deposited sequence and longer than the deposited sequence. Next, we constructed a candidate emesis gene set for further investigation in emesis related biology. We performed gene ontology enrichment on that candidate gene set which identified several calcium signaling terms as being enriched within this candidate gene set.

We also investigated enriched interacting proteins associated with these candidate emesis genes and identified additional calcium interacting molecules. Indeed, our current gene ontology enrichment data from Tables 2 (biologic process), 3 (molecular function), 4 (cellular compartment), 5 (interacting proteins with emesis), and 6 (Enriched UniProt Tissue Expression) demonstrate association of vomiting with Ca^2+^ and its related signals including: voltage calcium channel activity; the three isoforms of calmodulin (1, 2, and 3); calmodulin dependent protein kinase I (CAMKI), CAMK II (*α*, *β* and *γ* isoforms) and CAMK IV; Calcium release-activated calcium channel protein 1 (ORAI 1); stromal interaction molecule 1 (STIM1) protein; store-operated calcium entry; intracellular Ca^2+^-release channels (inositol triphosphate (IP_3_R)- and ryanodine (R_Y_R)-receptors) present in the membrane of the sarcoplasmic/endoplasmic reticulum (SER); and L-type calcium channels (LTCCs) subunits α1, 1C, 1D and 1F (Zhong et al., 2014a; Zhong et al., 2014b; Zhong et al., 2016; Zhong et al., 2018; Zhong et al., 2019; Darmani et al., 2014).

In line with our current RNAseq enrichment findings, we have previously proposed an overview of the involvement Ca^2+^ mobilization in the process of vomiting evoked by diverse emetogens (Zhong et al., 2017). First, selective (e.g., agonists of neurokinin NK_1_ (Miyano et al., 2010)-, serotonergic 5-HT_3_ (Hargreaves et al., 1996) -, dopaminergic D_2_ (Aman et al., 2007) - receptors) and non-specific (e.g., cisplatin) emetogens evoke vomiting *via* an increase in cytosolic Ca^2+^ concentration, which subsequently initiates downstream Ca^2+^-activated emetic signals. Second, cisplatin, one of the oldest and most widely used cancer chemotherapeutic (Alhadeff et al., 2017), also induces nausea and vomiting *via* Ca^2+^-dependent release of multiple neurotransmitters (including serotonin (5-HT), substance P (SP), dopamine, *etc.*) from both central emetic loci in the dorsal vagal complex (DVC) of the brainstem as well as peripherally in the GIT (Hesketh, 2008; Darmani and Ray, 2009). The DVC contains the emetic nuclei nucleus tractus solitarius (NTS), the dorsal motor nucleus of the vagus (DMNX) and the area postrema (AP). Moreover, we have confirmed direct involvement of Ca^2+^ ion-channels in emesis. Indeed, the term Ca^2+^-induced Ca^2+^-release (CICR), refers to a process where an initial extracellular Ca^2+^ influx *via* activation of plasma membrane voltage-operated Ca^2+^ channels trigger intracellular Ca^2+^ release from the SER Ca^2+^ stores, resulting in an increase in the cytosolic concentration of Ca^2+^ (Homma et al., 2006; Ziviani et al., 2011). In fact, the selective L-type Ca^2+^ channel (LTCC) agonist FPL64176 causes extracellular influx as well as vomiting in least shrews in a dose-dependent manner (Zhong et al., 2014a; Darmani et al., 2014). Moreover, the intracellular Ca^2+^ releasing agent thapsigargin [a selective SER Ca^2+^-ATPase (SERCA) inhibitor], evokes vomiting in least shrews by increasing the cytosolic concentration of Ca^2+^, initially *via* depletion of intracellular SER Ca^2+^-stores followed by store-operated extracellular Ca^2+^ entry (SOCE) through LTCCs and other plasma membrane bound calcium channels (Solovyova and Verkhratsky, 2003; Michelangeli and East, 2011; Beltran-Parrazal et al., 2012; Zhong et al., 2016). In the latter process both STIM one and ORAI1 proteins play essential roles, since the former polymerizes to form a tube-like structure connecting the SER to ORAI or LTCC or TRCP1, which act as the mouthpiece of calcium ion-channel in the cell membrane (Avila-Medina et al., 2016; Calderon-Sanchez et al., 2020). Intracellular Ca^2+^ release from the SER into the cytoplasm is mediated through inositol trisphosphate receptors (IP_3_Rs) and ryanodine receptors (RyRs) ion-channels present in the SER membrane (Gomez-Viquez et al., 2003). By evaluating the antiemetic effects of their respective antagonists (dantrolene and 2-APB), we have demonstrated that blockade of either of these intracellular Ca^2+^-release channels suppress vomiting evoked by diverse emetogens including FPL64176, thapsigargin, as well as selective agonists of 5-HT_3_R (2-methyl-serotonin) and NK_1_R (GR73632) (Zhong et al., 2014b; Zhong et al., 2016; Zhong et al., 2018; Zhong et al., 2019).

Tables 4–8 also highlight the role of cAMP-PKA signaling system in vomiting such as adenylate cyclase activity, cyclic AMP (cAMP) biosynthesis, phosphodiesterase (PDE) activity and activation of protein kinase A (PKA). In mammals cAMP is synthesized by 10 adenylate cyclase isoforms (Halls and Cooper, 2017). One of the best-studied second messenger molecules downstream of selected G-protein coupled receptors is cAMP. It is an example for a transient and diffusible second messenger which is involved in signal propagation by integrating multiple intracellular signaling pathways (Gancedo, 2013). cAMP activates PKA which results in phosphorylation of downstream intracellular signals. The emetic role of cAMP has been well established, since microinjection of cAMP analogs (e.g., 8-bromocAMP) or forskolin (to enhance endogenous levels of cAMP) in the brainstem DVC emetic locus area postrema, not only increases electrical activity of local neurons, but also induces vomiting in dogs (Carpenter et al., 1988). Moreover, administration of 8-chlorocAMP in cancer patients can evoke nausea and vomiting (Propper et al., 1999). Furthermore, phosphodiesterase inhibitors (PDEI) such as rolipram prevent cAMP metabolism and consequently increase cAMP tissue levels, which leads to excessive nausea and vomiting in humans and animals including least shrews (Mori et al., 2010; Alkam et al., 2014). In fact, one major side-effect of older PDEIs is excessive nausea and vomiting which often precludes their use in the clinical setting (Vanmierlo et al., 2016). PKA-phosphorylation is associated with peak vomit frequency during both immediate- and delayed-phases of vomiting caused by either cisplatin or cyclophosphamide in the least shrew (Darmani et al., 2013; Darmani et al., 2015; Alkam et al., 2014).

Other enriched intracellular emetic signals in the current investigation include CaMKII, ERK1/2 and PKC. Indeed, time-dependent Ca^2+^/calmodulin kinase IIα (CaMKIIα), protein kinase Cα/βII (PKCα/βII) and extracellular signal-regulated protein kinases 1 and 2 (ERK1/2) phosphorylation in the least shrew brainstem occurs following: i) 5-HT_3_R-evoked vomiting caused by its selective agonist 2-methyl-5-HT (Zhong et al., 2014b), ii) FPL64176 and thapsigargin-induced emesis in the least shrew (Zhong et al., 2016; Zhong et al., 2018), as well as iii) SP neurokinin NK_1_R-mediated vomiting evoked by the selective NK_1_R agonist GR73632 in the least shrew (Zhong et al., 2019). In addition, other published evidence demonstrates that phosphorylation of protein kinase Cα/βII (PKCα/βII) and ERK1/2 in least shrew brainstem are associated with cisplatin-induced emesis. In fact, significant upregulation of ERK1/2 phosphorylation occurs with peak vomit frequency during both the immediate and delayed phases of emesis caused by cisplatin in the least shrew (Darmani et al., 2013; Darmani et al., 2015).

As part of our analysis, we identified the set of conserved one-to-one orthologs among the shrew, human, mouse, dog, and ferret which resulted in 6,952 protein coding genes. We used a BLAST method to compare the sequence identity across human, mouse, dog, and ferret to shrew and in our analysis, we identified nucleotide identity to be lowest between shrew and mouse (85.9%) and similar (88.6%, 88.7%, and 88.9%; respectively) between shrew and human, ferret, and dog. Our analysis of protein coding showed similar results with shrew *versus* mouse having the lowest percent identity (89.9%) and shrew *versus* 91.45% (dog), 91.46% (ferret) and 91.89% (human) based on sequences obtained from the Ensembl database (Cunningham et al., 2019; Ferret Genome assembly: https://uswest.ensembl.org/Mustela_putorius_furo/Info/Index; Dog Genome assembly; Human Genome assembly: https://uswest.ensembl.org/Homo_sapiens/Info/Index; Mouse Genome assembly: http://uswest.ensembl.org/Mus_musculus/Info/Index).

The method used to identify the 16,720 orthologous protein coding genes relied upon a reciprocal best hit algorithm to map shrew transcripts to the human, mouse, ferret, and dog. Because of the requirement for reciprocal best hits, there are likely genes that are not found in our set of 1-to-1 orthologs. Although the reciprocal best hit approach is recognized as producing lower errors than some other methods (Moreno-Hagelsieb and Latimer, 2008), it has limitations when used in large gene families with differing paralogs across species, such as mammalian species (Dalquen and Dessimoz, 2013). Inability to identify a reciprocal best hit between large gene family members does not necessarily indicate absence of an orthologous gene, but rather it indicates absence of a reciprocal best hit relationships between the pair of genes being tested. Subsequently some members of large gene families, such as the L-type calcium channels are not fully represented in our set of protein coding orthologs. The calcium channel gene family is comprised of multiple paralogous gene members (Catterall 2011). The identification of multiple gene family members, such as voltage gated calcium ion-channels and phospholipase C, may offer insight into the specific family members involved in vomiting signaling cascades.

From among 6,952 protein coding conserved orthologs we performed a phenotype enrichment to see what phenotypes were enriched among the top 10% of the 6,952 genes. We identified many lethality and developmental phenotypes underscoring the role these genes play in development. Additionally, we identified phenotypes implicated in brain morphology and CNS synaptic transmission.

Finally, we performed a KEGG pathway enrichment to identify signaling pathways that were also enriched within the top 10% of conserved genes within the 6,952 genes. Our results show neurotransmitter signaling and memory associated pathways. We also identified pathways implicated in circadian entrainment and addiction. The identification of memory associated pathways associated with emesis is not surprising. Substance P is a an 11-amino acid neurotransmitter peptide that is well known for its role in emesis with additional roles implicated in modulating memory pathways (Graefe and Mohiuddin, 2022). Moreover, genes implicated in addiction and circadian cycle entrainment may play roles in the regulation or induction of emesis. For example, a connection between late chronotypes and chemotherapy-induced nausea and vomiting was identified in a recent study of risk factors associated with chemotherapy induced vomiting (Lee et al., 2017).

Our work provides a valuable resource for neurobiology, pharmacology, and neurophysiology in the shrew (Supplemental File 9, complete set of 16,720 shrew transcript sequences derived from brainstem and gut tissue). Our newly produced sequences greatly expand the genomic resources available for shrew and open the least shrew up as a model for neurobiology, memory, and addiction.

Some limitations of our study include the fact that we employed the blast algorithm to determine percent identity between shrew and other species. BLAST is a local alignment tool and could therefore produce higher than average identities in some regions compared to others (or between certain sequences compared to others). Additionally, our set of 16,720 shrew transcript sequences may include incomplete sequences due to low sequencing coverage in some regions of the transcriptome Furthermore, our data is lacking transcript sequences that were not expressed in the gut and brainstem. Nevertheless, this data is valuable, and provides a substantial genomics framework for investigating emesis in the least shrew.

## 5 Conclusion

We sequenced and analyzed shrew brainstem and gut transcriptomes associated with vomiting evoked by a substance P neurokinin NK_1_ receptor selective agonist and its interaction with a corresponding NK_1_ receptor selective antagonist. We discovered a set of 16,720 assembled transcripts that exhibit orthology to human, mouse, dog and/or ferret sequences and we identified a core set of emesis pathway signal transduction components. Together, these least shrew transcripts and associated analyses provide novel genomics resources for advancing emesis research (Figure 7) in both humans and other vomit-competent animal models of emesis.

## Data Availability

The datasets presented in this study can be found in online repositories. The names of the repository/repositories and accession number(s) can be found in the article/Supplementary Material.

## Supplementary material

Supplementary Table S1 Pairwise BLAST alignment between the previously deposited sequence for Tachykinin 1 Precursor (query sequence) versus RNA-seq generated Tachykinin 1 Precursor sequence (subject sequence). File contains URL for NCBI nucleotide entry for shrew TAC1 and two fasta seqs for TAC1 and the URL for the pairwise blast web site and a description of the parameters used to complete the blast. Supplementally, the document contains the BLAST alignment of the two sequences.

Supplementary Table S2 Pairwise BLAST alignment between the previously deposited sequence for Brain Derived Neurotrophic Factor (query sequence) versus RNA-seq generated BDNF sequence (subject sequence). File contains URL for NCBI nucleotide entry for shrew BDNF and two fasta seqs for BDNF and the URL for the pairwise blast web site and a description of the parameters used to complete the blast. Supplementally, the document contains the BLAST alignment of the two sequences.

Supplementary Table S3 Pairwise BLAST alignment between the previously deposited sequence for Brain Derived Neurotrophic Factor (query sequence) versus RNA-seq generated BDNF sequence (subject sequence). File contains URL for NCBI nucleotide entry for shrew BDNF and two fasta seqs for BDNF and the URL for the pairwise blast web site and a description of the parameters used to complete the blast. Supplementally, the document contains the BLAST alignment of the two sequences.

Supplementary Table S4 Set of 125 Least Shrew Emesis-Candidate Genes. File contains a table with the set of candidate genes including the gene symbol and the gene description.

Supplementary Table S5 Disease Genes Associations Among Shrew Candidate Emesis Gene Set. File contains a table with the subset of candidate genes that are associated with disease in the OMIM database.

Supplementary Table S6 Gene Ontology Enrichment for Candidate Emesis Genes – Biological Process (All Results). File contains a table with all the significant biological process terms in gene ontology enrichment analysis among the candidate genes.

Supplementary Table S7 Gene Ontology Enrichment for Candidate Emesis Genes – Molecular Function (All Results). File contains a table with all the significant molecular process terms in gene ontology enrichment analysis among the candidate genes.

Supplementary Table S8 Phenotype Enrichment of Top 10% Most Conserved Orthologs. File contains a set of enriched phenotypes with *p*-values and corrected p-values (FDR, Bonferroni) that are color coded by significance.

Supplementary Table S9 16,720 Shrew Transcript Sequences. File contains a set 16,720 shrew transcript sequences derived from brainstem and gut tissue that were assembled and mapped to orthologs.

Supplementary Table S10 Emesis Pathway and Best Matching Transcripts Across Species. File contains a set of 39 emesis pathway components and their best hits to transcripts from human, dog, mouse, ferret and a related shrew species (*Sorex Araneus*).
