# Supplementary material for: RNA sequencing least shrew (Cryptotis parva) brainstem and gut transcripts following administration of a selective substance P neurokinin NK1 receptor agonist and antagonist expands genomics resources for emesis research
Source: Front Genet. 2023 Feb 14;14:975087. doi: 10.3389/fgene.2023.975087 (PMC9972295; doi:10.3389/fgene.2023.975087)
Supplement: Supplementary file 1 [file Table1.DOCX]

**<https://www.ncbi.nlm.nih.gov/nuccore/224498024>**

FJ696706.1 Cryptotis parva Tac1 (PPT-A) mRNA, complete cds

**>TAC1ncbi**

**TGATGATCTCAGTTATTGGTCCGACTGGTCTGACAGCGACCAGATCAAGGAGGAGCTGCCTGAGCCCTTT**

**GAGCACGTTCTGCAGAGAATGGCCCGGAGACCCAAGTCTCAGCAGTTCTATGGATTGATGGGCAAACGTG**

**ATGCTGATTCCTCAATTGAAAAACAAATGGCCCTGCTAAAGGCTCTATATGGACATGGCCAGATTTCTCA**

**TAAAA**

**>TAC1rnaSeq**

**TCTTTCATTGGCCATTGACGCAAAGGAAGCTCAGTAAATAATACACTAAGTAGTTTATTTACGTCTTCTTTCATAATTCTGCATTGCACTCCTTCCGTAAGCCACAGAATTTAGAGCTCTTTTGCCCATTAGTCCAACAAAGGAATCTGTTTTATGTCTTTTATGAGAAATCTGGCCATGTCCATATAGAGCCTTTAGCAGGGCCATTTGTTTTTCAATTGAGGAATCAGCATCACGTTTGCCCATCAATCCATAGAACTGCTGAGACTTGGGTCTCCGGGCCATTCTCTGCAGAACGTGCTCAAAGGGCTCAGGCAGCTCCTCCTTGATCTGGTCGCTGTCAGACCAGTCGGACCAATAACTGAGATCATCATTGGTTCCCATCTCTTCTGCCAACAGTTGAGTGGAGGCGAGAAAAAAGACGGCTAATGCCACGAGGATTTTCATGTTGGAATTCCTAGAGTCCGGAAGCTCTGCAGATGCTCGACCAAGAGAGACATCGGCACCT**

<https://blast.ncbi.nlm.nih.gov/Blast.cgi?BLAST_SPEC=blast2seq&LINK_LOC=align2seq&PAGE_TYPE=BlastSearch>

Default parameters for “Highly similar sequences (megablast)”

**Query: TAC1ncbi Query ID: lcl|Query_20293 Length: 215**

**>TAC1rnaSeq**

**Sequence ID: Query_20295 Length: 508**

**Range 1: 159 to 373**

**Score:398 bits(215), Expect:1e-115,**

**Identities:215/215(100%), Gaps:0/215(0%), Strand: Plus/Minus**

**Query 1 TGATGATCTCAGTTATTGGTCCGACTGGTCTGACAGCGACCAGATCAAGGAGGAGCTGCC 60**

**||||||||||||||||||||||||||||||||||||||||||||||||||||||||||||**

**Sbjct 373 TGATGATCTCAGTTATTGGTCCGACTGGTCTGACAGCGACCAGATCAAGGAGGAGCTGCC 314**

**Query 61 TGAGCCCTTTGAGCACGTTCTGCAGAGAATGGCCCGGAGACCCAAGTCTCAGCAGTTCTA 120**

**||||||||||||||||||||||||||||||||||||||||||||||||||||||||||||**

**Sbjct 313 TGAGCCCTTTGAGCACGTTCTGCAGAGAATGGCCCGGAGACCCAAGTCTCAGCAGTTCTA 254**

**Query 121 TGGATTGATGGGCAAACGTGATGCTGATTCCTCAATTGAAAAACAAATGGCCCTGCTAAA 180**

**||||||||||||||||||||||||||||||||||||||||||||||||||||||||||||**

**Sbjct 253 TGGATTGATGGGCAAACGTGATGCTGATTCCTCAATTGAAAAACAAATGGCCCTGCTAAA 194**

**Query 181 GGCTCTATATGGACATGGCCAGATTTCTCATAAAA 215**

**|||||||||||||||||||||||||||||||||||**

**Sbjct 193 GGCTCTATATGGACATGGCCAGATTTCTCATAAAA 159**
