# Supplementary material for: RNA sequencing least shrew (Cryptotis parva) brainstem and gut transcripts following administration of a selective substance P neurokinin NK1 receptor agonist and antagonist expands genomics resources for emesis research
Source: Front Genet. 2023 Feb 14;14:975087. doi: 10.3389/fgene.2023.975087 (PMC9972295; doi:10.3389/fgene.2023.975087)
Supplement: Supplementary file 3 [file Table7.DOCX]

Gene Ontology Enrichment for Candidate Emesis Genes – Molecular Function (All Results)

See Table 3

| Term | Count | % | P-Value | Benjamini |
| --- | --- | --- | --- | --- |
| phosphatidylinositol phospholipase C activity | 14 | 11.5 | 7.90E-22 | 1.60E-19 |
| kinase activity | 21 | 17.2 | 2.60E-16 | 2.30E-14 |
| ATP binding | 43 | 35.2 | 5.70E-16 | 3.80E-14 |
| calmodulin binding | 19 | 15.6 | 8.10E-16 | 4.00E-14 |
| G-protein coupled serotonin receptor activity | 11 | 9 | 2.00E-15 | 8.20E-14 |
| phospholipase C activity | 9 | 7.4 | 5.20E-15 | 1.80E-13 |
| 1-phosphatidylinositol-3-kinase activity | 12 | 9.8 | 5.30E-15 | 1.50E-13 |
| calmodulin-dependent protein kinase activity | 10 | 8.2 | 1.20E-14 | 3.20E-13 |
| serotonin binding | 8 | 6.6 | 7.60E-14 | 1.70E-12 |
| neurotransmitter receptor activity | 10 | 8.2 | 7.60E-14 | 1.60E-12 |
| protein serine/threonine kinase activity | 22 | 18 | 1.30E-13 | 2.40E-12 |
| adenylate cyclase activity | 9 | 7.4 | 3.00E-13 | 5.10E-12 |
| phosphorus-oxygen lyase activity | 8 | 6.6 | 4.90E-13 | 7.80E-12 |
| 1-phosphatidylinositol-4-phosphate 3-kinase activity | 7 | 5.7 | 6.80E-13 | 9.90E-12 |
| signal transducer activity | 17 | 13.9 | 7.90E-13 | 1.10E-11 |
| protein kinase C activity | 8 | 6.6 | 3.90E-12 | 5.00E-11 |
| protein kinase activity | 17 | 13.9 | 3.60E-09 | 4.30E-08 |
| calcium ion binding | 23 | 18.9 | 3.60E-09 | 4.10E-08 |
| phosphatidylinositol 3-kinase activity | 5 | 4.1 | 1.10E-08 | 1.20E-07 |
| 1-phosphatidylinositol-3-kinase regulator activity | 5 | 4.1 | 3.30E-08 | 3.30E-07 |
| phosphatidylinositol-4,5-bisphosphate 3-kinase activity | 8 | 6.6 | 2.30E-07 | 2.20E-06 |
| inositol 1,4,5 trisphosphate binding | 5 | 4.1 | 7.00E-07 | 6.50E-06 |
| calcium-release channel activity | 5 | 4.1 | 7.00E-07 | 6.50E-06 |
| insulin receptor substrate binding | 4 | 3.3 | 5.10E-05 | 4.60E-04 |
| titin binding | 4 | 3.3 | 1.10E-04 | 9.50E-04 |
| ion channel binding | 7 | 5.7 | 1.30E-04 | 1.10E-03 |
| inositol 1,4,5-trisphosphate-sensitive calcium-release channel activity | 3 | 2.5 | 1.40E-04 | 1.10E-03 |
| N-terminal myristoylation domain binding | 3 | 2.5 | 1.40E-04 | 1.10E-03 |
| calcium-independent protein kinase C activity | 3 | 2.5 | 1.40E-04 | 1.10E-03 |
| Ras guanyl-nucleotide exchange factor activity | 7 | 5.7 | 1.50E-04 | 1.10E-03 |
| drug binding | 6 | 4.9 | 1.80E-04 | 1.30E-03 |
| phospholipase binding | 4 | 3.3 | 2.50E-04 | 1.70E-03 |
| calcium-dependent protein kinase C activity | 3 | 2.5 | 2.80E-04 | 1.90E-03 |
| ryanodine-sensitive calcium-release channel activity | 3 | 2.5 | 2.80E-04 | 1.90E-03 |
| calcium-induced calcium release activity | 3 | 2.5 | 2.80E-04 | 1.90E-03 |
| phosphatidylinositol 3-kinase regulator activity | 3 | 2.5 | 2.80E-04 | 1.90E-03 |
| phosphatidylinositol binding | 6 | 4.9 | 3.10E-04 | 2.00E-03 |
| protein phosphatase activator activity | 3 | 2.5 | 7.00E-04 | 4.50E-03 |
| nitric-oxide synthase regulator activity | 3 | 2.5 | 1.30E-03 | 8.00E-03 |
| calcium-transporting ATPase activity | 3 | 2.5 | 1.70E-03 | 1.00E-02 |
| inositol-1,4,5-trisphosphate 3-kinase activity | 3 | 2.5 | 2.10E-03 | 1.20E-02 |
| high voltage-gated calcium channel activity | 3 | 2.5 | 2.10E-03 | 1.20E-02 |
| voltage-gated calcium channel activity | 4 | 3.3 | 2.70E-03 | 1.50E-02 |
| phosphoric diester hydrolase activity | 3 | 2.5 | 3.00E-03 | 1.70E-02 |
| store-operated calcium channel activity | 3 | 2.5 | 3.00E-03 | 1.70E-02 |
| thioesterase binding | 3 | 2.5 | 4.10E-03 | 2.20E-02 |
| protein kinase binding | 9 | 7.4 | 4.70E-03 | 2.40E-02 |
| protein serine/threonine kinase activator activity | 3 | 2.5 | 6.80E-03 | 3.40E-02 |
| enzyme binding | 8 | 6.6 | 8.50E-03 | 4.20E-02 |
| calcium channel activity | 4 | 3.3 | 1.30E-02 | 6.00E-02 |
| tryptophan 5-monooxygenase activity | 2 | 1.6 | 1.40E-02 | 6.40E-02 |
| 1-(4-iodo-2,5-dimethoxyphenyl)propan-2-amine binding | 2 | 1.6 | 1.40E-02 | 6.40E-02 |
| histone kinase activity (H3-T6 specific) | 2 | 1.6 | 1.40E-02 | 6.40E-02 |
| calcium ion transmembrane transporter activity | 2 | 1.6 | 1.40E-02 | 6.40E-02 |
| calcium- and calmodulin-responsive adenylate cyclase activity | 2 | 1.6 | 1.40E-02 | 6.40E-02 |
| metal ion binding | 24 | 19.7 | 1.40E-02 | 6.50E-02 |
| calcium-dependent protein kinase inhibitor activity | 2 | 1.6 | 2.10E-02 | 9.10E-02 |
| tachykinin receptor activity | 2 | 1.6 | 2.10E-02 | 9.10E-02 |
| ligand-gated ion channel activity | 3 | 2.5 | 2.60E-02 | 1.10E-01 |
| voltage-gated calcium channel activity involved in cardiac muscle cell action potential | 2 | 1.6 | 2.70E-02 | 1.10E-01 |
| oxidoreductase activity, acting on paired donors, with incorporation or reduction of molecular oxygen, reduced pteridine as one donor, and incorporation of one atom of oxygen | 2 | 1.6 | 2.70E-02 | 1.10E-01 |
| receptor signaling protein activity | 3 | 2.5 | 3.10E-02 | 1.30E-01 |
| glutamate receptor binding | 2 | 1.6 | 3.40E-02 | 1.40E-01 |
| dopamine neurotransmitter receptor activity | 2 | 1.6 | 3.40E-02 | 1.40E-01 |
| nucleotide binding | 7 | 5.7 | 3.40E-02 | 1.30E-01 |
| neurotrophin TRKA receptor binding | 2 | 1.6 | 4.10E-02 | 1.50E-01 |
| adenylate cyclase binding | 2 | 1.6 | 6.10E-02 | 2.20E-01 |
| phosphatidylinositol 3-kinase regulatory subunit binding | 2 | 1.6 | 6.10E-02 | 2.20E-01 |
| dopamine binding | 2 | 1.6 | 6.70E-02 | 2.40E-01 |
| alpha-actinin binding | 2 | 1.6 | 8.60E-02 | 2.90E-01 |
| phosphotyrosine binding | 2 | 1.6 | 8.60E-02 | 2.90E-01 |
| nitric-oxide synthase binding | 2 | 1.6 | 9.30E-02 | 3.00E-01 |
| MAP kinase activity | 2 | 1.6 | 9.30E-02 | 3.00E-01 |
| GABA receptor binding | 2 | 1.6 | 9.30E-02 | 3.00E-01 |
