# Supplementary material for: RNA sequencing least shrew (Cryptotis parva) brainstem and gut transcripts following administration of a selective substance P neurokinin NK1 receptor agonist and antagonist expands genomics resources for emesis research
Source: Front Genet. 2023 Feb 14;14:975087. doi: 10.3389/fgene.2023.975087 (PMC9972295; doi:10.3389/fgene.2023.975087)
Supplement: Supplementary file 4 [file Table2.DOCX]

<https://www.ncbi.nlm.nih.gov/nuccore/1060647763>

>LC124902.1 Cryptotis parvus BDNF gene for brain-derived neurotrophic factor, partial cds, specimen_voucher: ASNHC:8192

**> BDNFncbi**

**ATTTCATACTTTGGTTGCATGAAGGCTGCCCCCATGAAAGAAGCAAACATCCGAGGACCAGGCAGCTTGG**

**CCTACCCAGGAGTGCGGACCCATGGGACTCTGGAGAGCGTGAATGGGCCCAAGGCAGGTTCACGAGGCCT**

**GACGTCATTGGCTGACACTTTTGAACACGTGATAGAAGAGCTGTTGGATGAGGACCAGAAAGTTCGGCCC**

**CATGAAGAAAACAATAAGGACGCGGACTTGTACACCTCCCGGGTGATGCTCAGTAGTCAAGTGCCTTTGG**

**AGCCTCCTCTTCTGTTTCTGCTGGAGGAATACAAAAATTACCTGGATGCTGCAAACATGTCGATGAGGGT**

**CCGGCGCCACTCTGACCCTGCCCGCCGTGGAGAGCTAAGCGTGTGCGACAGCATTAGCGAGTGGGTGACA**

**GCGGCAGATAAAAAGACTGCAGTGGACATGTCGGGCGGGACAGTCACTGTCCTTGAAAAAGTCCCCGTAT**

**CCAAAGGCCAACTGAAGCAGTACTTCTATGAGACCAAATGCAATCCCATGGGTTACACGAAGGAGGGCTG**

**CAGG**

**>BDNFrnaseq TRINITY_DN319153_c2_g4**

**ATTAAAGCAGCATGCAATTTATTATTATTATTATTATTATTTTTTAAACTTTTTACGTTTTCAATTCTTG**

**GCAACGGCAACAAACCACAACATTATCGAGGAATGTAATGCAGACTTTTTTTAAAGTTGTGCGCAAAATG**

**ACTGTTTCCCTTCTGGTCATGGATATGTCCAATAAATAGATTGTAGAACCACTGTACTGTATAAACTTCA**

**TTTATACATGCAGTTCATAAAATTATTTTTTCTTAACTGAATAATTTACCCTGTTATGTATATATACAAA**

**TAGATAATTTGTCTCAATATAATCTATACAACATAAATCCACTATCTTCCCCTCTTAATGGTCAATGTAC**

**ATACACAGGAAGTGTCTATCCTTATGAAGCGCCAGCCAATTCTCTTTTTGCTATCCATGGTAAGGGCCCG**

**CACGTACGACTGGGTAGTTCGGCACTGGGAATTCCAATGCCTTTTGTCTATGCCCCTGCAGCCCTCCTTC**

**GTGTAACCCATGGGATTGCATTTGGTCTCATAGAAGTACTGCTTCAGTTGGCCTTTGGATACGGGGACTT**

**TTTCAAGGACAGTGACTGTCCCGCCCGACATGTCCACTGCAGTCTTTTTATCTGCCGCTGTCACCCACTC**

**GCTAATGCTGTCGCACACGCTTAGCTCTCCACGGCGGGCAGGGTCAGAGTGGCGCCGGACCCTCATCGAC**

**ATGTTTGCAGCATCCAGGTAATTTTTGTATTCCTCCAGCAGAAACAGAAGAGGAGGCTCCAAAGGCACTT**

**GACTACTGAGCATCACCCGGGAGGTGTACAAGTCCGCGTCCTTATTGTTTTCTTCATGGGGCCGAACTTT**

**CTGGTCCTCATCCAACAGCTCTTCTATCACGTGTTCAAAAGTGTCAGCCAATGACGTCAGGCCTCGTGAA**

**CCTGCCTTGGGCCCATTCACGCTCTCCAGAGTCCCATGGGTCCGCACTCCTGGGTAGGCCAAGCTGCCTG**

**GTCCTCGGATGTTTGCTTCTTTCATGGGGGCAGCCTTCATGCAACCAAAGTATGAAATAACCATAGTAAG**

**GAAAAGGATGGTCATCACTCTTCTCACCTGGTGGAACTTCTTTGCGGCTTACACCACCCCGGTGGCTAGA**

**TCCTGTAGATAAACACTTGCATTTCCCAAAGTTAACCCAATATACCAACCCGGAGCTTGCCAAGAGTCTA**

**TTCCAGTCTACACCGCTAGGAAGCCAACTTCAGTAAGCTCAATGAGGGGACCAAACTGGGGCTCGCTTTT**

**CCAAACGTTCCGCTCCAAAATATGACTCTCTCCAGCCCCGATTTCAGTGTGATCCGAACCTTA**

<https://blast.ncbi.nlm.nih.gov/Blast.cgi?BLAST_SPEC=blast2seq&LINK_LOC=align2seq&PAGE_TYPE=BlastSearch>

Default parameters for “Highly similar sequences (megablast)”

Query: BDNFncbi Query ID: lcl|Query_11125 Length: 564

>BDNFrnaseq TRINITY_DN319153_c2_g4

Sequence ID: Query_11127 Length: 1323

Range 1: 475 to 1038

Score:1042 bits(564), Expect:0.0,

Identities:564/564(100%), Gaps:0/564(0%), Strand: Plus/Minus

Query 1 ATTTCATACTTTGGTTGCATGAAGGCTGCCCCCATGAAAGAAGCAAACATCCGAGGACCA 60

||||||||||||||||||||||||||||||||||||||||||||||||||||||||||||

Sbjct 1038 ATTTCATACTTTGGTTGCATGAAGGCTGCCCCCATGAAAGAAGCAAACATCCGAGGACCA 979

Query 61 GGCAGCTTGGCCTACCCAGGAGTGCGGACCCATGGGACTCTGGAGAGCGTGAATGGGCCC 120

||||||||||||||||||||||||||||||||||||||||||||||||||||||||||||

Sbjct 978 GGCAGCTTGGCCTACCCAGGAGTGCGGACCCATGGGACTCTGGAGAGCGTGAATGGGCCC 919

Query 121 AAGGCAGGTTCACGAGGCCTGACGTCATTGGCTGACACTTTTGAACACGTGATAGAAGAG 180

||||||||||||||||||||||||||||||||||||||||||||||||||||||||||||

Sbjct 918 AAGGCAGGTTCACGAGGCCTGACGTCATTGGCTGACACTTTTGAACACGTGATAGAAGAG 859

Query 181 CTGTTGGATGAGGACCAGAAAGTTCGGCCCCATGAAGAAAACAATAAGGACGCGGACTTG 240

||||||||||||||||||||||||||||||||||||||||||||||||||||||||||||

Sbjct 858 CTGTTGGATGAGGACCAGAAAGTTCGGCCCCATGAAGAAAACAATAAGGACGCGGACTTG 799

Query 241 TACACCTCCCGGGTGATGCTCAGTAGTCAAGTGCCTTTGGAGCCTCCTCTTCTGTTTCTG 300

||||||||||||||||||||||||||||||||||||||||||||||||||||||||||||

Sbjct 798 TACACCTCCCGGGTGATGCTCAGTAGTCAAGTGCCTTTGGAGCCTCCTCTTCTGTTTCTG 739

Query 301 CTGGAGGAATACAAAAATTACCTGGATGCTGCAAACATGTCGATGAGGGTCCGGCGCCAC 360

||||||||||||||||||||||||||||||||||||||||||||||||||||||||||||

Sbjct 738 CTGGAGGAATACAAAAATTACCTGGATGCTGCAAACATGTCGATGAGGGTCCGGCGCCAC 679

Query 361 TCTGACCCTGCCCGCCGTGGAGAGCTAAGCGTGTGCGACAGCATTAGCGAGTGGGTGACA 420

||||||||||||||||||||||||||||||||||||||||||||||||||||||||||||

Sbjct 678 TCTGACCCTGCCCGCCGTGGAGAGCTAAGCGTGTGCGACAGCATTAGCGAGTGGGTGACA 619

Query 421 GCGGCAGATAAAAAGACTGCAGTGGACATGTCGGGCGGGACAGTCACTGTCCTTGAAAAA 480

||||||||||||||||||||||||||||||||||||||||||||||||||||||||||||

Sbjct 618 GCGGCAGATAAAAAGACTGCAGTGGACATGTCGGGCGGGACAGTCACTGTCCTTGAAAAA 559

Query 481 GTCCCCGTATCCAAAGGCCAACTGAAGCAGTACTTCTATGAGACCAAATGCAATCCCATG 540

||||||||||||||||||||||||||||||||||||||||||||||||||||||||||||

Sbjct 558 GTCCCCGTATCCAAAGGCCAACTGAAGCAGTACTTCTATGAGACCAAATGCAATCCCATG 499

Query 541 GGTTACACGAAGGAGGGCTGCAGG 564

||||||||||||||||||||||||

Sbjct 498 GGTTACACGAAGGAGGGCTGCAGG 475
