# Supplementary material for: RNA sequencing least shrew (Cryptotis parva) brainstem and gut transcripts following administration of a selective substance P neurokinin NK1 receptor agonist and antagonist expands genomics resources for emesis research
Source: Front Genet. 2023 Feb 14;14:975087. doi: 10.3389/fgene.2023.975087 (PMC9972295; doi:10.3389/fgene.2023.975087)
Supplement: Supplementary file 5 [file Table5.DOCX]

| Gene | Gene Name | OMIM Disease |
| --- | --- | --- |
| ADCY1 | adenylate cyclase 1(ADCY1) | Deafness, autosomal recessive 44, |
| ADCY5 | adenylate cyclase 5(ADCY5) | Dyskinesia, familial, with facial myokymia, |
| ADCY6 | adenylate cyclase 6(ADCY6) | Lethal congenital contracture syndrome 8, |
| ATP2A1 | ATPase sarcoplasmic/endoplasmic reticulum Ca2+ transporting 1(ATP2A1) | Brody myopathy, |
| ATP2A2 | ATPase sarcoplasmic/endoplasmic reticulum Ca2+ transporting 2(ATP2A2) | Acrokeratosis verruciformis, Darier disease, |
| CACNA1C | calcium voltage-gated channel subunit alpha1 C(CACNA1C) | Timothy syndrome, Brugada syndrome 3, |
| CACNA1D | calcium voltage-gated channel subunit alpha1 D(CACNA1D) | Sinoatrial node dysfunction and deafness, Primary aldosteronism, seizures, and neurologic abnormalities, |
| CACNA1F | calcium voltage-gated channel subunit alpha1 F(CACNA1F) | Night blindness, congenital stationary (incomplete), 2A, X-linked, Cone-rod dystrophy, X-linked, 3, Aland Island eye disease, |
| CALM1 | calmodulin 1(CALM1) | Ventricular tachycardia, catecholaminergic polymorphic, 4, Long QT syndrome 14, |
| CALM2 | calmodulin 2(CALM2) | Long QT syndrome 15, |
| DBH | dopamine beta-hydroxylase(DBH) | Dopamine beta-hydroxylase deficiency, Dopamine-beta-hydroxylase activity levels, plasma, |
| HTR1A | 5-hydroxytryptamine receptor 1A(HTR1A) | Periodic fever, menstrual cycle dependent, |
| HTR2A | 5-hydroxytryptamine receptor 2A(HTR2A) | Alcohol dependence, susceptibility to, Obsessive-compulsive disorder, susceptibility to, Schizophrenia, susceptibility to, Anorexia nervosa, susceptibility to, Major depressive disorder, response to citalopram therapy in, Seasonal affective disorder, susceptibility to, |
| ITPR1 | inositol 1,4,5-trisphosphate receptor type 1(ITPR1) | Spinocerebellar ataxia 29, congenital nonprogressive, Spinocerebellar ataxia 15, |
| ITPR2 | inositol 1,4,5-trisphosphate receptor type 2(ITPR2) | Anhidrosis, isolated, with normal sweat glands, |
| ITPR3 | inositol 1,4,5-trisphosphate receptor type 3(ITPR3) | Diabetes, type 1, susceptibility to, |
| PIK3CA | phosphatidylinositol-4,5-bisphosphate 3-kinase catalytic subunit alpha(PIK3CA) | Breast cancer, somatic, Colorectal cancer, somatic, Hepatocellular carcinoma, somatic, Nevus, epidermal, somatic, Ovarian cancer, somatic, Keratosis, seborrheic, somatic, Nonsmall cell lung cancer, somatic, Megalencephaly-capillary malformation-polymicrogyria syndrome, somatic, CLOVE syndrome, somatic, Gastric cancer, somatic, Cowden syndrome 5, |
| PIK3CD | phosphatidylinositol-4,5-bisphosphate 3-kinase catalytic subunit delta(PIK3CD) | Immunodeficiency 14, |
| PIK3R1 | phosphoinositide-3-kinase regulatory subunit 1(PIK3R1) | SHORT syndrome, Agammaglobulinemia 7, autosomal recessive, Immunodeficiency 36, |
| PIK3R2 | phosphoinositide-3-kinase regulatory subunit 2(PIK3R2) | Megalencephaly-polymicrogyria-polydactyly-hydrocephalus syndrome 1, |
| PIK3R5 | phosphoinositide-3-kinase regulatory subunit 5(PIK3R5) | Ataxia-oculomotor apraxia 3, |
| PLCB1 | phospholipase C beta 1(PLCB1) | Epileptic encephalopathy, early infantile, 12, |
| PLCB2 | phospholipase C beta 2(PLCB2) | Platelet PLC beta-2 deficiency, |
| PLCB4 | phospholipase C beta 4(PLCB4) | Auriculocondylar syndrome 2, |
| PLCD1 | phospholipase C delta 1(PLCD1) | Nail disorder, nonsyndromic congenital, 3, (leukonychia), |
| PLCE1 | phospholipase C epsilon 1(PLCE1) | Nephrotic syndrome, type 3, |
| PLCG2 | phospholipase C gamma 2(PLCG2) | Familial cold autoinflammatory syndrome 3, Autoinflammation, antibody deficiency, and immune dysregulation syndrome, |
| PRKCA | protein kinase C alpha(PRKCA) | Pituitary tumor, invasive, |
| PRKCD | protein kinase C delta(PRKCD) | Autoimmune lymphoproliferative syndrome, type III, |
| PRKCG | protein kinase C gamma(PRKCG) | Spinocerebellar ataxia 14, |
| PRKCH | protein kinase C eta(PRKCH) | Cerebral infarction, susceptibility to, |
| RYR1 | ryanodine receptor 1(RYR1) | Central core disease, Neuromuscular disease, congenital, with uniform type 1 fiber, King-Denborough syndrome, Malignant hyperthermia susceptibility 1, Minicore myopathy with external ophthalmoplegia, |
| RYR2 | ryanodine receptor 2(RYR2) | Arrhythmogenic right ventricular dysplasia 2, Ventricular tachycardia, catecholaminergic polymorphic, 1, |
| SLC6A4 | solute carrier family 6 member 4(SLC6A4) | Obsessive-compulsive disorder, Anxiety-related personality traits, |
| TPH2 | tryptophan hydroxylase 2(TPH2) | Unipolar depression, susceptibility to, Attention deficit-hyperactivity disorder, susceptibility to, 7, |
