# Supplementary material for: RNA sequencing least shrew (Cryptotis parva) brainstem and gut transcripts following administration of a selective substance P neurokinin NK1 receptor agonist and antagonist expands genomics resources for emesis research
Source: Front Genet. 2023 Feb 14;14:975087. doi: 10.3389/fgene.2023.975087 (PMC9972295; doi:10.3389/fgene.2023.975087)
Supplement: Supplementary file 6 [file Table3.DOCX]

**<https://www.ncbi.nlm.nih.gov/nuccore/409968179>**

JQ715623.1 Cryptotis parva neurokinin 1 receptor (NK1R) mRNA, partial cds

**>NK1R-ncbi**

**TCGTGGTGACCTCCGTAGTGGGCAATGTGGTGGTGATGTGGATCATCTTGGCCCACAAGAGAATGAGGAC**

**AGTGACCAACTATTTTCTGGTGAACCTGGCCTTCGCCGAGGCCTCTATGGCGGCGTTCAACACGGTGGTG**

**AACTTCACCTACGCCGTCCACAACGAGTGGTACTACGGTCTGTTCTACTGCAAGTTCCACAACTTCTTCC**

**CCATCGCTGCTGTCTTCGCTAGCATCTACTCCATGACGGCCGTGGCCTTTGACAGATACATGGCCATCAT**

**CCATCCCCTCCAGCCCCGACTGTCAGCCACAGCCACCAAGGTGGTTATCTTGGTCATCTGGGTCCTGGCT**

**CTCCTTCTGGCCTTCCCCCAGGGCTACTACTCAACCACAGAGACTCTGCCCAACAGAGTGGTGTGCCGGA**

**TTGAGTGGCCAGAACATCCCAACCAGATTTATGAGAAAGTGTACCACATCAGTGTGACTGTGCTGGTCTA**

**TTTCCTCCCGCTGCTGGTTATTGGCTATGCATACACCGTCGTGGGAATCACCCTGTGGGCCAGCGAGATC**

**CCTGGGGATTCCTCTGACCGATACCATGATCAAGTCTCTGCC**

**>NK1R-rnaSeq**

**GGAGGTCAGGTCCAGGGATGAGGGTGTGGCCTTGGGGCCATCCTCCAGCTCCTCCTCCTGAGCCCCTACCA**

**CAGTGGACACAGTGGTCTCGAGGCGGCTGACCTTATACACACTGCCCTGCGTCTGGAGGTATCTGGTGGAT**

**TTCATTTCGAGCCCCTCATAGTCACCGGCACTGATGAAGGGGCAGCACCGGAAGGCATGCTTGAAGCCCAC**

**CCGGAACCTGTCATTCAGGCAGCAGTAGATGATGGGGTTGTACATAGTGGAGCTCATGGCCAGCCACATGA**

**TGGCCAGGTAAACCTGCTGAATAAACTTCTCTGAGGTGAGGTTCGGCTTGATGTAGGGCAGGAGGAAGAAG**

**ATGTGGAAGGGCAGCCAGCAGATGGCAAAGGTGCAGACCACCACAATCATCATCTTGACCACCTTTCGCTT**

**GGCAGAGACTTGATCATGGTATCGGTCAGAGGAATCCCCAGGGATCTCGCTGGCCCACAGGGTGATTCCCA**

**CGACGGTGTATGCATAGCCAATAACCAGCAGCGGGAGGAAATAGACCAGCACAGTCACACTGATGTGGTAC**

**ACTTTCTCATAAATCTGGTTGGGATGTTCTGGCCACTCAATCCGGCACACCACTCTGTTGGGCAGAGTCTC**

**TGTGGTTGAGTAGTAGCCCTGGGGGAAGGCCAGAAGGAGAGCCAGGACCCAGATGACCAAGATAACCACCT**

**TGGTGGCTGTGGCTGACAGTCGGGGCTGGAGGGGATGGATGATGGCCATGTATCTGTCAAAGGCCACGGCC**

**GTCATGGAGTAGATGCTAGCGAAGACAGCAGCGATGGGGAAGAAGTTGTGGAACTTGCAGTAGAACAGACC**

**GTAGTACCACTCGTTGTGGACGGCGTAGGTGAAGTTCACCACCGTGTTGAACGCCGCCATAGAGGCCTCGG**

**CGAAGGCCAGGTTCACCAGAAAATAGTTGGTCACTGTCCTCATTCTCTTGTGGGCCAAGATGATCCACATC**

**ACCACCACATTGCCCACTACGGAGGTCACCACGATGACCGTGTAGGCAGTGGCCCAGAGGACAATTTGCCA**

**GGCGGGCTGTACGAACTGGTTGGACTCCGAGGTGTTGGTGGACACGTTTGGGAAAAGGTCTGTGTCCACGG**

**GGAGCACGTCATCCATTTCTCTGCCGAGCGGTGAAGCCTGTGTTCAACTACCACCTTTGCAGCAAGGTTCT**

**GAGGCAGGAGCCTGGGGTCTTAGGGGGGCTGGGAGAGGGGGTGTCAGAGTCCTGTTTAACCGAGACCAAAG**

**CACAGGAGGCGCTTTCTGCACCCGGTTCCCTCGCAAACCGGAGACCTGGTGCCCAGGACAGAAACACGTCT**

**AGAGGAGCTGTGCCTCCCTCGTGCCTCTGAATTCCTCCACTGCTCAGCTTCTAGGCGCATCTGAGTTCAGG**

**TATCTGAAGACAGTGAGTCTTCGGCGTTGGGACTGGAGAAGCAGGAAAATTCCACGGGTTACGGTTTCAGG**

**AAGCGGAGACCCCCGGCGCCCCGCACCTGCTGCTGCTTGCAACTCTCCCTCGCCCAGCCTGCAGCAGGGAA**

**AGGCAAACGCCGGCC**

[**https://blast.ncbi.nlm.nih.gov/Blast.cgi?BLAST_SPEC=blast2seq&LINK_LOC=align2seq&PAGE_TYPE=BlastSearch**](https://blast.ncbi.nlm.nih.gov/Blast.cgi?BLAST_SPEC=blast2seq&LINK_LOC=align2seq&PAGE_TYPE=BlastSearch)

Default parameters for “Highly similar sequences (megablast)”

**Query: NK1R-ncbi Query ID: lcl|Query_29381 Length: 602**

**>NK1R-rnaSeq**

**Sequence ID: Query_29383 Length: 1577**

**Range 1: 427 to 1028**

**Score:1112 bits(602), Expect:0.0,**

**Identities:602/602(100%), Gaps:0/602(0%), Strand: Plus/Minus**

**Query 1 TCGTGGTGACCTCCGTAGTGGGCAATGTGGTGGTGATGTGGATCATCTTGGCCCACAAGA 60**

**||||||||||||||||||||||||||||||||||||||||||||||||||||||||||||**

**Sbjct 1028 TCGTGGTGACCTCCGTAGTGGGCAATGTGGTGGTGATGTGGATCATCTTGGCCCACAAGA 969**

**Query 61 GAATGAGGACAGTGACCAACTATTTTCTGGTGAACCTGGCCTTCGCCGAGGCCTCTATGG 120**

**||||||||||||||||||||||||||||||||||||||||||||||||||||||||||||**

**Sbjct 968 GAATGAGGACAGTGACCAACTATTTTCTGGTGAACCTGGCCTTCGCCGAGGCCTCTATGG 909**

**Query 121 CGGCGTTCAACACGGTGGTGAACTTCACCTACGCCGTCCACAACGAGTGGTACTACGGTC 180**

**||||||||||||||||||||||||||||||||||||||||||||||||||||||||||||**

**Sbjct 908 CGGCGTTCAACACGGTGGTGAACTTCACCTACGCCGTCCACAACGAGTGGTACTACGGTC 849**

**Query 181 TGTTCTACTGCAAGTTCCACAACTTCTTCCCCATCGCTGCTGTCTTCGCTAGCATCTACT 240**

**||||||||||||||||||||||||||||||||||||||||||||||||||||||||||||**

**Sbjct 848 TGTTCTACTGCAAGTTCCACAACTTCTTCCCCATCGCTGCTGTCTTCGCTAGCATCTACT 789**

**Query 241 CCATGACGGCCGTGGCCTTTGACAGATACATGGCCATCATCCATCCCCTCCAGCCCCGAC 300**

**||||||||||||||||||||||||||||||||||||||||||||||||||||||||||||**

**Sbjct 788 CCATGACGGCCGTGGCCTTTGACAGATACATGGCCATCATCCATCCCCTCCAGCCCCGAC 729**

**Query 301 TGTCAGCCACAGCCACCAAGGTGGTTATCTTGGTCATCTGGGTCCTGGCTCTCCTTCTGG 360**

**||||||||||||||||||||||||||||||||||||||||||||||||||||||||||||**

**Sbjct 728 TGTCAGCCACAGCCACCAAGGTGGTTATCTTGGTCATCTGGGTCCTGGCTCTCCTTCTGG 669**

**Query 361 CCTTCCCCCAGGGCTACTACTCAACCACAGAGACTCTGCCCAACAGAGTGGTGTGCCGGA 420**

**||||||||||||||||||||||||||||||||||||||||||||||||||||||||||||**

**Sbjct 668 CCTTCCCCCAGGGCTACTACTCAACCACAGAGACTCTGCCCAACAGAGTGGTGTGCCGGA 609**

**Query 421 TTGAGTGGCCAGAACATCCCAACCAGATTTATGAGAAAGTGTACCACATCAGTGTGACTG 480**

**||||||||||||||||||||||||||||||||||||||||||||||||||||||||||||**

**Sbjct 608 TTGAGTGGCCAGAACATCCCAACCAGATTTATGAGAAAGTGTACCACATCAGTGTGACTG 549**

**Query 481 TGCTGGTCTATTTCCTCCCGCTGCTGGTTATTGGCTATGCATACACCGTCGTGGGAATCA 540**

**||||||||||||||||||||||||||||||||||||||||||||||||||||||||||||**

**Sbjct 548 TGCTGGTCTATTTCCTCCCGCTGCTGGTTATTGGCTATGCATACACCGTCGTGGGAATCA 489**

**Query 541 CCCTGTGGGCCAGCGAGATCCCTGGGGATTCCTCTGACCGATACCATGATCAAGTCTCTG 600**

**||||||||||||||||||||||||||||||||||||||||||||||||||||||||||||**

**Sbjct 488 CCCTGTGGGCCAGCGAGATCCCTGGGGATTCCTCTGACCGATACCATGATCAAGTCTCTG 429**

**Query 601 CC 602**

**||**

**Sbjct 428 CC 427**
