# Supplementary material for: RNA sequencing least shrew (Cryptotis parva) brainstem and gut transcripts following administration of a selective substance P neurokinin NK1 receptor agonist and antagonist expands genomics resources for emesis research
Source: Front Genet. 2023 Feb 14;14:975087. doi: 10.3389/fgene.2023.975087 (PMC9972295; doi:10.3389/fgene.2023.975087)
Supplement: Supplementary file 7 [file Table4.DOCX]

| Gene Name | Gene Description |
| --- | --- |
| ADCY1 | adenylate cyclase 1 [Source:HGNC Symbol;Acc:HGNC:232] |
| ADCY2 | adenylate cyclase 2 [Source:HGNC Symbol;Acc:HGNC:233] |
| ADCY3 | adenylate cyclase 3 [Source:HGNC Symbol;Acc:HGNC:234] |
| ADCY4 | adenylate cyclase 4 [Source:HGNC Symbol;Acc:HGNC:235] |
| ADCY5 | adenylate cyclase 5 [Source:HGNC Symbol;Acc:HGNC:236] |
| ADCY6 | adenylate cyclase 6 [Source:HGNC Symbol;Acc:HGNC:237] |
| ADCY7 | adenylate cyclase 7 [Source:HGNC Symbol;Acc:HGNC:238] |
| ADCY8 | adenylate cyclase 8 [Source:HGNC Symbol;Acc:HGNC:239] |
| ADCY9 | adenylate cyclase 9 [Source:HGNC Symbol;Acc:HGNC:240] |
| ADCYAP1 | adenylate cyclase activating polypeptide 1 [Source:HGNC Symbol;Acc:HGNC:241] |
| ATP2A1 (SERCA1) | ATPase sarcoplasmic/endoplasmic reticulum Ca2+ transporting 1 [Source:HGNC Symbol;Acc:HGNC:811] |
| ATP2A2 (SERCA2) | ATPase sarcoplasmic/endoplasmic reticulum Ca2+ transporting 2 [Source:HGNC Symbol;Acc:HGNC:812] |
| ATP2A3 (SERCA3) | ATPase sarcoplasmic/endoplasmic reticulum Ca2+ transporting 3 [Source:HGNC Symbol;Acc:HGNC:813] |
| CACNA1C (LTCC) | calcium voltage-gated channel subunit alpha1 C [Source:HGNC Symbol;Acc:HGNC:1390] |
| CACNA1D (LTCC) | calcium voltage-gated channel subunit alpha1 D [Source:HGNC Symbol;Acc:HGNC:1391] |
| CACNA1F (LTCC) | calcium voltage-gated channel subunit alpha1 F [Source:HGNC Symbol;Acc:HGNC:1393] |
| CALM1 | calmodulin 1 [Source:HGNC Symbol;Acc:HGNC:1442] |
| CALM2 | calmodulin 2 [Source:HGNC Symbol;Acc:HGNC:1445] |
| CALM3 | calmodulin 3 [Source:HGNC Symbol;Acc:HGNC:1449] |
| CAMK1 | calcium/calmodulin dependent protein kinase I [Source:HGNC Symbol;Acc:HGNC:1459] |
| CAMK1D | calcium/calmodulin dependent protein kinase ID [Source:HGNC Symbol;Acc:HGNC:19341] |
| CAMK1G | calcium/calmodulin dependent protein kinase IG [Source:HGNC Symbol;Acc:HGNC:14585] |
| CAMK2A (CaKiiA) | calcium/calmodulin dependent protein kinase II alpha [Source:HGNC Symbol;Acc:HGNC:1460] |
| CAMK2B | calcium/calmodulin dependent protein kinase II beta [Source:HGNC Symbol;Acc:HGNC:1461] |
| CAMK2D | calcium/calmodulin dependent protein kinase II delta [Source:HGNC Symbol;Acc:HGNC:1462] |
| CAMK2G | calcium/calmodulin dependent protein kinase II gamma [Source:HGNC Symbol;Acc:HGNC:1463] |
| CAMK2N1 | calcium/calmodulin dependent protein kinase II inhibitor 1 [Source:HGNC Symbol;Acc:HGNC:24190] |
| CAMK2N2 | calcium/calmodulin dependent protein kinase II inhibitor 2 [Source:HGNC Symbol;Acc:HGNC:24197] |
| CAMK4 | calcium/calmodulin dependent protein kinase IV [Source:HGNC Symbol;Acc:HGNC:1464] |
| CAMKK1 | calcium/calmodulin dependent protein kinase kinase 1 [Source:HGNC Symbol;Acc:HGNC:1469] |
| CAMKK2 | calcium/calmodulin dependent protein kinase kinase 2 [Source:HGNC Symbol;Acc:HGNC:1470] |
| CAMKMT | calmodulin-lysine N-methyltransferase [Source:HGNC Symbol;Acc:HGNC:26276] |
| CAMKV | CaM kinase like vesicle associated [Source:HGNC Symbol;Acc:HGNC:28788] |
| CNR1 | cannabinoid receptor 1 [Source:HGNC Symbol;Acc:HGNC:2159] |
| CNRIP1 | cannabinoid receptor interacting protein 1 [Source:HGNC Symbol;Acc:HGNC:24546] |
| CRACR2A (SOCE) | calcium release activated channel regulator 2A [Source:HGNC Symbol;Acc:HGNC:28657] |
| CRACR2B (SOCE) | calcium release activated channel regulator 2B [Source:HGNC Symbol;Acc:HGNC:28703] |
| DBH | dopamine beta-hydroxylase [Source:HGNC Symbol;Acc:HGNC:2689] |
| DRD1 | dopamine receptor D1 [Source:HGNC Symbol;Acc:HGNC:3020] |
| DRD2 | dopamine receptor D2 [Source:HGNC Symbol;Acc:HGNC:3023] |
| GAREM1 | GRB2 associated regulator of MAPK1 (ERK2) subtype 1 [Source:HGNC Symbol;Acc:HGNC:26136] |
| GAREM2 | GRB2 associated regulator of MAPK1 (ERK2) subtype 2 [Source:HGNC Symbol;Acc:HGNC:27172] |
| HTR1A | 5-hydroxytryptamine receptor 1A [Source:HGNC Symbol;Acc:HGNC:5286] |
| HTR1B | 5-hydroxytryptamine receptor 1B [Source:HGNC Symbol;Acc:HGNC:5287] |
| HTR1D | 5-hydroxytryptamine receptor 1D [Source:HGNC Symbol;Acc:HGNC:5289] |
| HTR1E | 5-hydroxytryptamine receptor 1E [Source:HGNC Symbol;Acc:HGNC:5291] |
| HTR1F | 5-hydroxytryptamine receptor 1F [Source:HGNC Symbol;Acc:HGNC:5292] |
| HTR2A | 5-hydroxytryptamine receptor 2A [Source:HGNC Symbol;Acc:HGNC:5293] |
| HTR2B | 5-hydroxytryptamine receptor 2B [Source:HGNC Symbol;Acc:HGNC:5294] |
| HTR2C | 5-hydroxytryptamine receptor 2C [Source:HGNC Symbol;Acc:HGNC:5295] |
| HTR3B | 5-hydroxytryptamine receptor 3B [Source:HGNC Symbol;Acc:HGNC:5298] |
| HTR4 | 5-hydroxytryptamine receptor 4 [Source:HGNC Symbol;Acc:HGNC:5299] |
| HTR6 | 5-hydroxytryptamine receptor 6 [Source:HGNC Symbol;Acc:HGNC:5301] |
| HTR7 | 5-hydroxytryptamine receptor 7 [Source:HGNC Symbol;Acc:HGNC:5302] |
| ITPR1 | inositol 1,4,5-trisphosphate receptor type 1 [Source:HGNC Symbol;Acc:HGNC:6180] |
| ITPR2 | inositol 1,4,5-trisphosphate receptor type 2 [Source:HGNC Symbol;Acc:HGNC:6181] |
| ITPR3 | inositol 1,4,5-trisphosphate receptor type 3 [Source:HGNC Symbol;Acc:HGNC:6182] |
| ITPRIP | inositol 1,4,5-trisphosphate receptor interacting protein [Source:HGNC Symbol;Acc:HGNC:29370] |
| MAPK1 (ERK2) | mitogen-activated protein kinase 1 [Source:HGNC Symbol;Acc:HGNC:6871] |
| MAPK3 (ERK1) | mitogen-activated protein kinase 3 [Source:HGNC Symbol;Acc:HGNC:6877] |
| NYAP1 | neuronal tyrosine phosphorylated phosphoinositide-3-kinase adaptor 1 [Source:HGNC Symbol;Acc:HGNC:22009] |
| NYAP2 | neuronal tyrosine-phosphorylated phosphoinositide-3-kinase adaptor 2 [Source:HGNC Symbol;Acc:HGNC:29291] |
| ORAI2 | ORAI calcium release-activated calcium modulator 2 [Source:HGNC Symbol;Acc:HGNC:21667] |
| ORAI3 | ORAI calcium release-activated calcium modulator 3 [Source:HGNC Symbol;Acc:HGNC:28185] |
| PDPK1 | 3-phosphoinositide dependent protein kinase 1 [Source:HGNC Symbol;Acc:HGNC:8816] |
| PIK3AP1 | phosphoinositide-3-kinase adaptor protein 1 [Source:HGNC Symbol;Acc:HGNC:30034] |
| PIK3C2A | phosphatidylinositol-4-phosphate 3-kinase catalytic subunit type 2 alpha [Source:HGNC Symbol;Acc:HGNC:8971] |
| PIK3C2B | phosphatidylinositol-4-phosphate 3-kinase catalytic subunit type 2 beta [Source:HGNC Symbol;Acc:HGNC:8972] |
| PIK3C2G | phosphatidylinositol-4-phosphate 3-kinase catalytic subunit type 2 gamma [Source:HGNC Symbol;Acc:HGNC:8973] |
| PIK3C3 | phosphatidylinositol 3-kinase catalytic subunit type 3 [Source:HGNC Symbol;Acc:HGNC:8974] |
| PIK3CA | phosphatidylinositol-4,5-bisphosphate 3-kinase catalytic subunit alpha [Source:HGNC Symbol;Acc:HGNC:8975] |
| PIK3CB | phosphatidylinositol-4,5-bisphosphate 3-kinase catalytic subunit beta [Source:HGNC Symbol;Acc:HGNC:8976] |
| PIK3CD | phosphatidylinositol-4,5-bisphosphate 3-kinase catalytic subunit delta [Source:HGNC Symbol;Acc:HGNC:8977] |
| PIK3CG | phosphatidylinositol-4,5-bisphosphate 3-kinase catalytic subunit gamma [Source:HGNC Symbol;Acc:HGNC:8978] |
| PIK3IP1 | phosphoinositide-3-kinase interacting protein 1 [Source:HGNC Symbol;Acc:HGNC:24942] |
| PIK3R1 | phosphoinositide-3-kinase regulatory subunit 1 [Source:HGNC Symbol;Acc:HGNC:8979] |
| PIK3R1 | phosphoinositide-3-kinase regulatory subunit 1 [Source:HGNC Symbol;Acc:HGNC:8979] |
| PIK3R2 | phosphoinositide-3-kinase regulatory subunit 2 [Source:HGNC Symbol;Acc:HGNC:8980] |
| PIK3R3 | phosphoinositide-3-kinase regulatory subunit 3 [Source:HGNC Symbol;Acc:HGNC:8981] |
| PIK3R4 | phosphoinositide-3-kinase regulatory subunit 4 [Source:HGNC Symbol;Acc:HGNC:8982] |
| PIK3R5 | phosphoinositide-3-kinase regulatory subunit 5 [Source:HGNC Symbol;Acc:HGNC:30035] |
| PIK3R6 | phosphoinositide-3-kinase regulatory subunit 6 [Source:HGNC Symbol;Acc:HGNC:27101] |
| PIRT | phosphoinositide interacting regulator of transient receptor potential channels [Source:HGNC Symbol;Acc:HGNC:37239] |
| PLCB1 | phospholipase C beta 1 [Source:HGNC Symbol;Acc:HGNC:15917] |
| PLCB2 | phospholipase C beta 2 [Source:HGNC Symbol;Acc:HGNC:9055] |
| PLCB3 | phospholipase C beta 3 [Source:HGNC Symbol;Acc:HGNC:9056] |
| PLCB4 | phospholipase C beta 4 [Source:HGNC Symbol;Acc:HGNC:9059] |
| PLCD1 | phospholipase C delta 1 [Source:HGNC Symbol;Acc:HGNC:9060] |
| PLCD3 | phospholipase C delta 3 [Source:HGNC Symbol;Acc:HGNC:9061] |
| PLCD4 | phospholipase C delta 4 [Source:HGNC Symbol;Acc:HGNC:9062] |
| PLCE1 | phospholipase C epsilon 1 [Source:HGNC Symbol;Acc:HGNC:17175] |
| PLCG1 | phospholipase C gamma 1 [Source:HGNC Symbol;Acc:HGNC:9065] |
| PLCG2 | phospholipase C gamma 2 [Source:HGNC Symbol;Acc:HGNC:9066] |
| PLCH1 | phospholipase C eta 1 [Source:HGNC Symbol;Acc:HGNC:29185] |
| PLCH2 | phospholipase C eta 2 [Source:HGNC Symbol;Acc:HGNC:29037] |
| PLCL1 | phospholipase C like 1 (inactive) [Source:HGNC Symbol;Acc:HGNC:9063] |
| PLCL2 | phospholipase C like 2 [Source:HGNC Symbol;Acc:HGNC:9064] |
| PLCXD2 | phosphatidylinositol specific phospholipase C X domain containing 2 [Source:HGNC Symbol;Acc:HGNC:26462] |
| PLCXD3 | phosphatidylinositol specific phospholipase C X domain containing 3 [Source:HGNC Symbol;Acc:HGNC:31822] |
| PRKCA | protein kinase C alpha [Source:HGNC Symbol;Acc:HGNC:9393] |
| PRKCB | protein kinase C beta [Source:HGNC Symbol;Acc:HGNC:9395] |
| PRKCD | protein kinase C delta [Source:HGNC Symbol;Acc:HGNC:9399] |
| PRKCE | protein kinase C epsilon [Source:HGNC Symbol;Acc:HGNC:9401] |
| PRKCG | protein kinase C gamma [Source:HGNC Symbol;Acc:HGNC:9402] |
| PRKCH | protein kinase C eta [Source:HGNC Symbol;Acc:HGNC:9403] |
| PRKCI | protein kinase C iota [Source:HGNC Symbol;Acc:HGNC:9404] |
| PRKCQ | protein kinase C theta [Source:HGNC Symbol;Acc:HGNC:9410] |
| RYR1 | ryanodine receptor 1 [Source:HGNC Symbol;Acc:HGNC:10483] |
| RYR2 | ryanodine receptor 2 [Source:HGNC Symbol;Acc:HGNC:10484] |
| RYR3 | ryanodine receptor 3 [Source:HGNC Symbol;Acc:HGNC:10485] |
| SARAF | store-operated calcium entry associated regulatory factor [Source:HGNC Symbol;Acc:HGNC:28789] |
| SLC6A4 | solute carrier family 6 member 4 [Source:HGNC Symbol;Acc:HGNC:11050] |
| SPSB1 | splA/ryanodine receptor domain and SOCS box containing 1 [Source:HGNC Symbol;Acc:HGNC:30628] |
| SPSB2 | splA/ryanodine receptor domain and SOCS box containing 2 [Source:HGNC Symbol;Acc:HGNC:29522] |
| SPSB4 | splA/ryanodine receptor domain and SOCS box containing 4 [Source:HGNC Symbol;Acc:HGNC:30630] |
| TAC1 | tachykinin precursor 1 [Source:HGNC Symbol;Acc:HGNC:11517] |
| TACR1 | tachykinin receptor 1 [Source:HGNC Symbol;Acc:HGNC:11526] |
| TACR2 | tachykinin receptor 2 [Source:HGNC Symbol;Acc:HGNC:11527] |
| TPH1 | tryptophan hydroxylase 1 [Source:HGNC Symbol;Acc:HGNC:12008] |
| TPH2 | tryptophan hydroxylase 2 [Source:HGNC Symbol;Acc:HGNC:20692] |
| TRPC1 | transient receptor potential cation channel subfamily C member 1 [Source:HGNC Symbol;Acc:HGNC:12333] |
| WIPI1 | WD repeat domain, phosphoinositide interacting 1 [Source:HGNC Symbol;Acc:HGNC:25471] |
| WIPI2 | WD repeat domain, phosphoinositide interacting 2 [Source:HGNC Symbol;Acc:HGNC:32225] |
