# Supplementary material for: RNA sequencing least shrew (Cryptotis parva) brainstem and gut transcripts following administration of a selective substance P neurokinin NK1 receptor agonist and antagonist expands genomics resources for emesis research
Source: Front Genet. 2023 Feb 14;14:975087. doi: 10.3389/fgene.2023.975087 (PMC9972295; doi:10.3389/fgene.2023.975087)
Supplement: Supplementary file 8 [file Table6.DOCX]

Gene Ontology Enrichment for Candidate Emesis Genes– Biological Process (All Results)

See Table 2 for subset of results

| Term | Count | % | P-Value | Benjamini |
| --- | --- | --- | --- | --- |
| intracellular signal transduction | 34 | 27.9 | 2.70E-26 | 2.50E-23 |
| release of sequestered calcium ion into cytosol | 16 | 13.1 | 1.10E-22 | 5.00E-20 |
| platelet activation | 20 | 16.4 | 3.70E-21 | 1.10E-18 |
| activation of adenylate cyclase activity | 15 | 12.3 | 6.40E-21 | 1.50E-18 |
| adenylate cyclase-inhibiting G-protein coupled receptor signaling pathway | 15 | 12.3 | 9.00E-20 | 1.70E-17 |
| phosphatidylinositol biosynthetic process | 15 | 12.3 | 2.50E-18 | 3.90E-16 |
| inositol phosphate metabolic process | 14 | 11.5 | 5.90E-18 | 7.80E-16 |
| lipid catabolic process | 16 | 13.1 | 2.30E-17 | 2.70E-15 |
| protein phosphorylation | 26 | 21.3 | 9.50E-16 | 1.00E-13 |
| phosphatidylinositol 3-kinase signaling | 11 | 9 | 1.50E-15 | 1.50E-13 |
| calcium ion transport | 14 | 11.5 | 5.40E-15 | 4.60E-13 |
| serotonin receptor signaling pathway | 9 | 7.4 | 5.80E-15 | 4.50E-13 |
| phosphatidylinositol-3-phosphate biosynthetic process | 12 | 9.8 | 3.00E-14 | 2.10E-12 |
| chemical synaptic transmission | 19 | 15.6 | 7.20E-14 | 4.80E-12 |
| cAMP biosynthetic process | 9 | 7.4 | 1.10E-13 | 6.70E-12 |
| activation of protein kinase A activity | 9 | 7.4 | 1.90E-13 | 1.10E-11 |
| cyclic nucleotide biosynthetic process | 8 | 6.6 | 5.40E-13 | 3.00E-11 |
| peptidyl-serine phosphorylation | 14 | 11.5 | 4.10E-12 | 2.10E-10 |
| cAMP-mediated signaling | 10 | 8.2 | 4.20E-12 | 2.10E-10 |
| phosphatidylinositol-mediated signaling | 13 | 10.7 | 1.00E-11 | 4.80E-10 |
| renal water homeostasis | 9 | 7.4 | 4.30E-11 | 1.90E-09 |
| phosphatidylinositol phosphorylation | 12 | 9.8 | 5.40E-11 | 2.30E-09 |
| adenylate cyclase-activating G-protein coupled receptor signaling pathway | 10 | 8.2 | 6.00E-11 | 2.50E-09 |
| calcium-mediated signaling | 10 | 8.2 | 7.30E-11 | 2.80E-09 |
| regulation of cardiac conduction | 10 | 8.2 | 1.80E-10 | 6.60E-09 |
| cellular response to glucagon stimulus | 9 | 7.4 | 3.00E-10 | 1.10E-08 |
| G-protein coupled receptor signaling pathway, coupled to cyclic nucleotide second messenger | 9 | 7.4 | 9.80E-10 | 3.40E-08 |
| Fc-epsilon receptor signaling pathway | 13 | 10.7 | 4.50E-09 | 1.50E-07 |
| phospholipase C-activating G-protein coupled receptor signaling pathway | 9 | 7.4 | 1.90E-08 | 6.20E-07 |
| regulation of phosphatidylinositol 3-kinase activity | 5 | 4.1 | 8.00E-08 | 2.50E-06 |
| vasoconstriction | 6 | 4.9 | 9.10E-08 | 2.70E-06 |
| calcium ion transmembrane transport | 10 | 8.2 | 1.60E-07 | 4.50E-06 |
| cellular response to forskolin | 5 | 4.1 | 1.60E-07 | 4.50E-06 |
| regulation of cardiac muscle contraction by regulation of the release of sequestered calcium ion | 6 | 4.9 | 1.70E-07 | 4.60E-06 |
| Fc-gamma receptor signaling pathway involved in phagocytosis | 10 | 8.2 | 2.70E-07 | 7.30E-06 |
| regulation of behavior | 5 | 4.1 | 2.80E-07 | 7.40E-06 |
| Wnt signaling pathway, calcium modulating pathway | 7 | 5.7 | 2.90E-07 | 7.20E-06 |
| cellular calcium ion homeostasis | 9 | 7.4 | 2.90E-07 | 7.10E-06 |
| T cell receptor signaling pathway | 10 | 8.2 | 9.90E-07 | 2.40E-05 |
| response to amphetamine | 6 | 4.9 | 2.30E-06 | 5.40E-05 |
| epidermal growth factor receptor signaling pathway | 7 | 5.7 | 2.60E-06 | 5.90E-05 |
| memory | 7 | 5.7 | 4.70E-06 | 1.10E-04 |
| regulation of insulin secretion | 7 | 5.7 | 7.50E-06 | 1.60E-04 |
| negative regulation of glial cell apoptotic process | 4 | 3.3 | 1.20E-05 | 2.50E-04 |
| regulation of cytokinesis | 5 | 4.1 | 1.50E-05 | 3.20E-04 |
| ion transmembrane transport | 10 | 8.2 | 1.70E-05 | 3.50E-04 |
| regulation of phosphatidylinositol 3-kinase signaling | 7 | 5.7 | 1.80E-05 | 3.60E-04 |
| store-operated calcium entry | 4 | 3.3 | 1.80E-05 | 3.60E-04 |
| regulation of cell communication by electrical coupling involved in cardiac conduction | 4 | 3.3 | 1.80E-05 | 3.60E-04 |
| response to morphine | 5 | 4.1 | 1.90E-05 | 3.50E-04 |
| activation of phospholipase C activity | 5 | 4.1 | 3.10E-05 | 5.70E-04 |
| positive regulation of ERK1 and ERK2 cascade | 9 | 7.4 | 3.30E-05 | 6.00E-04 |
| long-term memory | 5 | 4.1 | 4.20E-05 | 7.50E-04 |
| response to calcium ion | 6 | 4.9 | 5.40E-05 | 9.40E-04 |
| inositol phosphate-mediated signaling | 4 | 3.3 | 5.40E-05 | 9.30E-04 |
| phosphatidylinositol metabolic process | 4 | 3.3 | 5.40E-05 | 9.30E-04 |
| response to drug | 11 | 9 | 5.70E-05 | 9.60E-04 |
| regulation of synaptic transmission, GABAergic | 4 | 3.3 | 7.10E-05 | 1.20E-03 |
| phosphorylation | 7 | 5.7 | 7.40E-05 | 1.20E-03 |
| regulation of heart rate | 5 | 4.1 | 8.10E-05 | 1.30E-03 |
| MAPK cascade | 10 | 8.2 | 9.70E-05 | 1.50E-03 |
| detection of calcium ion | 4 | 3.3 | 1.20E-04 | 1.80E-03 |
| positive regulation of cyclic nucleotide metabolic process | 3 | 2.5 | 1.50E-04 | 2.20E-03 |
| signal transduction | 21 | 17.2 | 1.60E-04 | 2.40E-03 |
| regulation of cellular response to heat | 6 | 4.9 | 1.80E-04 | 2.70E-03 |
| protein autophosphorylation | 8 | 6.6 | 2.10E-04 | 3.10E-03 |
| response to hypoxia | 8 | 6.6 | 2.10E-04 | 3.10E-03 |
| response to pain | 4 | 3.3 | 2.10E-04 | 3.10E-03 |
| regulation of release of sequestered calcium ion into cytosol by sarcoplasmic reticulum | 4 | 3.3 | 2.60E-04 | 3.60E-03 |
| positive regulation of phosphatidylinositol biosynthetic process | 3 | 2.5 | 2.90E-04 | 4.10E-03 |
| intestine smooth muscle contraction | 3 | 2.5 | 2.90E-04 | 4.10E-03 |
| regulation of ryanodine-sensitive calcium-release channel activity | 4 | 3.3 | 3.00E-04 | 4.10E-03 |
| positive regulation of cytosolic calcium ion concentration | 7 | 5.7 | 3.70E-04 | 4.90E-03 |
| positive regulation of neuron projection development | 6 | 4.9 | 4.10E-04 | 5.40E-03 |
| positive regulation of protein dephosphorylation | 4 | 3.3 | 4.10E-04 | 5.40E-03 |
| regulation of cardiac muscle contraction | 4 | 3.3 | 4.10E-04 | 5.40E-03 |
| positive regulation of nitric-oxide synthase activity | 4 | 3.3 | 4.70E-04 | 6.10E-03 |
| positive regulation of cyclic-nucleotide phosphodiesterase activity | 3 | 2.5 | 4.80E-04 | 6.20E-03 |
| sensory perception of pain | 5 | 4.1 | 4.80E-04 | 6.10E-03 |
| positive regulation of MAP kinase activity | 5 | 4.1 | 7.80E-04 | 9.70E-03 |
| positive regulation of peptidyl-threonine phosphorylation | 4 | 3.3 | 8.70E-04 | 1.10E-02 |
| positive regulation of release of sequestered calcium ion into cytosol | 4 | 3.3 | 8.70E-04 | 1.10E-02 |
| regulation of high voltage-gated calcium channel activity | 3 | 2.5 | 1.00E-03 | 1.20E-02 |
| behavioral fear response | 4 | 3.3 | 1.10E-03 | 1.30E-02 |
| regulation of rhodopsin mediated signaling pathway | 4 | 3.3 | 1.10E-03 | 1.30E-02 |
| tachykinin receptor signaling pathway | 3 | 2.5 | 1.30E-03 | 1.60E-02 |
| cellular response to caffeine | 3 | 2.5 | 1.30E-03 | 1.60E-02 |
| interferon-gamma-mediated signaling pathway | 5 | 4.1 | 1.60E-03 | 1.80E-02 |
| leukocyte migration | 6 | 4.9 | 1.70E-03 | 1.90E-02 |
| negative regulation of synaptic transmission, glutamatergic | 3 | 2.5 | 1.70E-03 | 1.90E-02 |
| positive regulation of ryanodine-sensitive calcium-release channel activity | 3 | 2.5 | 1.70E-03 | 1.90E-02 |
| positive regulation of protein serine/threonine kinase activity | 4 | 3.3 | 1.90E-03 | 2.10E-02 |
| circadian rhythm | 5 | 4.1 | 1.90E-03 | 2.10E-02 |
| positive regulation of GTPase activity | 12 | 9.8 | 2.10E-03 | 2.30E-02 |
| adenylate cyclase-modulating G-protein coupled receptor signaling pathway | 4 | 3.3 | 2.20E-03 | 2.40E-02 |
| peptidyl-threonine phosphorylation | 4 | 3.3 | 2.40E-03 | 2.50E-02 |
| protein lipidation | 3 | 2.5 | 2.60E-03 | 2.70E-02 |
| protein localization to pre-autophagosomal structure | 3 | 2.5 | 2.60E-03 | 2.70E-02 |
| response to toxic substance | 5 | 4.1 | 3.00E-03 | 3.10E-02 |
| negative regulation of ryanodine-sensitive calcium-release channel activity | 3 | 2.5 | 3.10E-03 | 3.20E-02 |
| G-protein coupled receptor internalization | 3 | 2.5 | 3.10E-03 | 3.20E-02 |
| positive regulation of phosphoprotein phosphatase activity | 3 | 2.5 | 3.60E-03 | 3.70E-02 |
| positive regulation of glucose import in response to insulin stimulus | 3 | 2.5 | 3.60E-03 | 3.70E-02 |
| regulation of dopamine secretion | 3 | 2.5 | 3.60E-03 | 3.70E-02 |
| cellular response to ethanol | 3 | 2.5 | 4.20E-03 | 4.20E-02 |
| calcium ion transport into cytosol | 3 | 2.5 | 4.20E-03 | 4.20E-02 |
| negative regulation of peptidyl-threonine phosphorylation | 3 | 2.5 | 4.20E-03 | 4.20E-02 |
| behavioral response to cocaine | 3 | 2.5 | 4.20E-03 | 4.20E-02 |
| endoplasmic reticulum calcium ion homeostasis | 3 | 2.5 | 4.80E-03 | 4.80E-02 |
| toll-like receptor 9 signaling pathway | 3 | 2.5 | 4.80E-03 | 4.80E-02 |
| positive regulation of telomere capping | 3 | 2.5 | 5.50E-03 | 5.30E-02 |
| B cell receptor signaling pathway | 4 | 3.3 | 6.50E-03 | 6.20E-02 |
| muscle contraction | 5 | 4.1 | 6.90E-03 | 6.50E-02 |
| response to corticosterone | 3 | 2.5 | 7.00E-03 | 6.50E-02 |
| smooth muscle contraction | 3 | 2.5 | 7.00E-03 | 6.50E-02 |
| labyrinthine layer blood vessel development | 3 | 2.5 | 7.70E-03 | 7.10E-02 |
| temperature homeostasis | 3 | 2.5 | 7.70E-03 | 7.10E-02 |
| glycogen catabolic process | 3 | 2.5 | 9.40E-03 | 8.50E-02 |
| positive regulation of protein autophosphorylation | 3 | 2.5 | 9.40E-03 | 8.50E-02 |
| gamma-aminobutyric acid signaling pathway | 3 | 2.5 | 1.00E-02 | 9.20E-02 |
| ERK1 and ERK2 cascade | 3 | 2.5 | 1.20E-02 | 1.10E-01 |
| cytokine production | 3 | 2.5 | 1.30E-02 | 1.10E-01 |
| natural killer cell chemotaxis | 2 | 1.6 | 1.40E-02 | 1.20E-01 |
| histone H3-T6 phosphorylation | 2 | 1.6 | 1.40E-02 | 1.20E-01 |
| phospholipase C-activating serotonin receptor signaling pathway | 2 | 1.6 | 1.40E-02 | 1.20E-01 |
| positive regulation of endoplasmic reticulum calcium ion concentration | 2 | 1.6 | 1.40E-02 | 1.20E-01 |
| operant conditioning | 2 | 1.6 | 1.40E-02 | 1.20E-01 |
| regulation of skeletal muscle adaptation | 2 | 1.6 | 1.40E-02 | 1.20E-01 |
| vascular endothelial growth factor receptor signaling pathway | 4 | 3.3 | 1.40E-02 | 1.20E-01 |
| regulation of nitric-oxide synthase activity | 3 | 2.5 | 1.40E-02 | 1.20E-01 |
| positive regulation of calcium ion transport | 3 | 2.5 | 1.40E-02 | 1.20E-01 |
| positive regulation of NF-kappaB transcription factor activity | 5 | 4.1 | 1.40E-02 | 1.20E-01 |
| response to endoplasmic reticulum stress | 4 | 3.3 | 1.60E-02 | 1.30E-01 |
| macroautophagy | 4 | 3.3 | 1.60E-02 | 1.30E-01 |
| positive regulation of DNA binding | 3 | 2.5 | 1.60E-02 | 1.30E-01 |
| positive regulation of cytosolic calcium ion concentration involved in phospholipase C-activating G-protein coupled signaling pathway | 3 | 2.5 | 1.60E-02 | 1.30E-01 |
| cellular response to insulin stimulus | 4 | 3.3 | 1.70E-02 | 1.40E-01 |
| positive regulation of telomerase activity | 3 | 2.5 | 1.80E-02 | 1.40E-01 |
| negative regulation of insulin receptor signaling pathway | 3 | 2.5 | 1.80E-02 | 1.40E-01 |
| insulin receptor signaling pathway | 4 | 3.3 | 1.80E-02 | 1.40E-01 |
| positive regulation of establishment of protein localization to plasma membrane | 3 | 2.5 | 1.90E-02 | 1.40E-01 |
| protein kinase C-activating G-protein coupled receptor signaling pathway | 3 | 2.5 | 2.00E-02 | 1.50E-01 |
| adaptive immune response | 5 | 4.1 | 2.10E-02 | 1.50E-01 |
| angiogenesis | 6 | 4.9 | 2.10E-02 | 1.50E-01 |
| MAPK import into nucleus | 2 | 1.6 | 2.10E-02 | 1.60E-01 |
| regulation of relaxation of cardiac muscle | 2 | 1.6 | 2.10E-02 | 1.60E-01 |
| adenylate cyclase-inhibiting serotonin receptor signaling pathway | 2 | 1.6 | 2.10E-02 | 1.60E-01 |
| cardiac neural crest cell development involved in heart development | 2 | 1.6 | 2.10E-02 | 1.60E-01 |
| negative regulation of dopamine secretion | 2 | 1.6 | 2.10E-02 | 1.60E-01 |
| serotonin biosynthetic process | 2 | 1.6 | 2.10E-02 | 1.60E-01 |
| respiratory burst involved in defense response | 2 | 1.6 | 2.10E-02 | 1.60E-01 |
| regulation of cytosolic calcium ion concentration | 3 | 2.5 | 2.10E-02 | 1.60E-01 |
| lipopolysaccharide-mediated signaling pathway | 3 | 2.5 | 2.10E-02 | 1.60E-01 |
| response to cocaine | 3 | 2.5 | 2.10E-02 | 1.60E-01 |
| positive regulation of telomere maintenance via telomerase | 3 | 2.5 | 2.10E-02 | 1.60E-01 |
| locomotory behavior | 4 | 3.3 | 2.10E-02 | 1.60E-01 |
| positive regulation of epithelial cell migration | 3 | 2.5 | 2.20E-02 | 1.60E-01 |
| cellular response to epidermal growth factor stimulus | 3 | 2.5 | 2.20E-02 | 1.60E-01 |
| lipid metabolic process | 5 | 4.1 | 2.50E-02 | 1.80E-01 |
| regulation of heart rate by cardiac conduction | 3 | 2.5 | 2.50E-02 | 1.80E-01 |
| response to epidermal growth factor | 2 | 1.6 | 2.80E-02 | 1.90E-01 |
| detection of abiotic stimulus | 2 | 1.6 | 2.80E-02 | 1.90E-01 |
| regulation of Golgi inheritance | 2 | 1.6 | 2.80E-02 | 1.90E-01 |
| neutrophil extravasation | 2 | 1.6 | 2.80E-02 | 1.90E-01 |
| feeding behavior | 3 | 2.5 | 2.90E-02 | 2.00E-01 |
| long-term synaptic potentiation | 3 | 2.5 | 2.90E-02 | 2.00E-01 |
| regulation of G-protein coupled receptor protein signaling pathway | 3 | 2.5 | 3.10E-02 | 2.10E-01 |
| adenylate cyclase-inhibiting dopamine receptor signaling pathway | 2 | 1.6 | 3.50E-02 | 2.30E-01 |
| regulation of hormone secretion | 2 | 1.6 | 3.50E-02 | 2.30E-01 |
| regulation of synapse structural plasticity | 2 | 1.6 | 3.50E-02 | 2.30E-01 |
| caveolin-mediated endocytosis | 2 | 1.6 | 3.50E-02 | 2.30E-01 |
| cellular response to temperature stimulus | 2 | 1.6 | 3.50E-02 | 2.30E-01 |
| sarcoplasmic reticulum calcium ion transport | 2 | 1.6 | 3.50E-02 | 2.30E-01 |
| indolalkylamine biosynthetic process | 2 | 1.6 | 3.50E-02 | 2.30E-01 |
| regulation of dopamine uptake involved in synaptic transmission | 2 | 1.6 | 3.50E-02 | 2.30E-01 |
| activation of MAPK activity | 4 | 3.3 | 4.00E-02 | 2.60E-01 |
| visual learning | 3 | 2.5 | 4.00E-02 | 2.60E-01 |
| cardiac conduction | 3 | 2.5 | 4.00E-02 | 2.60E-01 |
| activation of protein kinase activity | 3 | 2.5 | 4.00E-02 | 2.60E-01 |
| positive regulation of cell migration | 5 | 4.1 | 4.10E-02 | 2.60E-01 |
| regulation of platelet aggregation | 2 | 1.6 | 4.10E-02 | 2.60E-01 |
| activation of store-operated calcium channel activity | 2 | 1.6 | 4.10E-02 | 2.60E-01 |
| positive regulation of receptor binding | 2 | 1.6 | 4.10E-02 | 2.60E-01 |
| trachea formation | 2 | 1.6 | 4.10E-02 | 2.60E-01 |
| sensory perception of bitter taste | 2 | 1.6 | 4.10E-02 | 2.60E-01 |
| cerebral cortex GABAergic interneuron migration | 2 | 1.6 | 4.10E-02 | 2.60E-01 |
| positive regulation of fat cell differentiation | 3 | 2.5 | 4.30E-02 | 2.70E-01 |
| substantia nigra development | 3 | 2.5 | 4.60E-02 | 2.90E-01 |
| positive regulation of angiogenesis | 4 | 3.3 | 4.70E-02 | 2.90E-01 |
| regulation of peptidyl-serine phosphorylation | 2 | 1.6 | 4.80E-02 | 2.90E-01 |
| positive regulation of B cell receptor signaling pathway | 2 | 1.6 | 4.80E-02 | 2.90E-01 |
| regulation of cardiac muscle contraction by calcium ion signaling | 2 | 1.6 | 4.80E-02 | 2.90E-01 |
| aromatic amino acid family metabolic process | 2 | 1.6 | 4.80E-02 | 2.90E-01 |
| regulation of store-operated calcium entry | 2 | 1.6 | 4.80E-02 | 2.90E-01 |
| negative regulation of fibroblast apoptotic process | 2 | 1.6 | 4.80E-02 | 2.90E-01 |
| peristalsis | 2 | 1.6 | 4.80E-02 | 2.90E-01 |
| cellular response to catecholamine stimulus | 2 | 1.6 | 4.80E-02 | 2.90E-01 |
| release of sequestered calcium ion into cytosol by sarcoplasmic reticulum | 2 | 1.6 | 4.80E-02 | 2.90E-01 |
| cellular response to calcium ion | 3 | 2.5 | 5.00E-02 | 3.00E-01 |
| inflammatory response | 7 | 5.7 | 5.10E-02 | 3.00E-01 |
| G-protein coupled receptor signaling pathway | 12 | 9.8 | 5.10E-02 | 3.00E-01 |
| cardiac muscle hypertrophy | 2 | 1.6 | 5.50E-02 | 3.20E-01 |
| regulation of early endosome to late endosome transport | 2 | 1.6 | 5.50E-02 | 3.20E-01 |
| positive regulation of growth hormone secretion | 2 | 1.6 | 5.50E-02 | 3.20E-01 |
| regulation of cardiac muscle cell action potential involved in regulation of contraction | 2 | 1.6 | 5.50E-02 | 3.20E-01 |
| negative regulation of sodium ion transmembrane transporter activity | 2 | 1.6 | 5.50E-02 | 3.20E-01 |
| T cell chemotaxis | 2 | 1.6 | 5.50E-02 | 3.20E-01 |
| regulation of stress-activated MAPK cascade | 2 | 1.6 | 5.50E-02 | 3.20E-01 |
| phospholipid metabolic process | 3 | 2.5 | 5.50E-02 | 3.20E-01 |
| positive regulation of cardiac muscle cell apoptotic process | 2 | 1.6 | 6.20E-02 | 3.50E-01 |
| outer ear morphogenesis | 2 | 1.6 | 6.20E-02 | 3.50E-01 |
| Bergmann glial cell differentiation | 2 | 1.6 | 6.20E-02 | 3.50E-01 |
| positive regulation of adenylate cyclase activity involved in G-protein coupled receptor signaling pathway | 2 | 1.6 | 6.20E-02 | 3.50E-01 |
| protein oligomerization | 3 | 2.5 | 6.30E-02 | 3.50E-01 |
| negative regulation of neuron apoptotic process | 4 | 3.3 | 6.60E-02 | 3.60E-01 |
| cell cycle | 5 | 4.1 | 6.70E-02 | 3.60E-01 |
| regulation of appetite | 2 | 1.6 | 6.80E-02 | 3.70E-01 |
| negative regulation of cytosolic calcium ion concentration | 2 | 1.6 | 6.80E-02 | 3.70E-01 |
| positive regulation of glial cell proliferation | 2 | 1.6 | 6.80E-02 | 3.70E-01 |
| negative regulation of potassium ion transport | 2 | 1.6 | 6.80E-02 | 3.70E-01 |
| regulation of dopamine metabolic process | 2 | 1.6 | 6.80E-02 | 3.70E-01 |
| phospholipase C-activating dopamine receptor signaling pathway | 2 | 1.6 | 6.80E-02 | 3.70E-01 |
| positive regulation of acute inflammatory response | 2 | 1.6 | 6.80E-02 | 3.70E-01 |
| G2/M transition of mitotic cell cycle | 4 | 3.3 | 7.20E-02 | 3.80E-01 |
| bone remodeling | 2 | 1.6 | 7.50E-02 | 3.90E-01 |
| striatum development | 2 | 1.6 | 7.50E-02 | 3.90E-01 |
| behavioral response to ethanol | 2 | 1.6 | 7.50E-02 | 3.90E-01 |
| dopamine metabolic process | 2 | 1.6 | 7.50E-02 | 3.90E-01 |
| regulation of ossification | 2 | 1.6 | 7.50E-02 | 3.90E-01 |
| regulation of ventricular cardiac muscle cell action potential | 2 | 1.6 | 7.50E-02 | 3.90E-01 |
| response to caffeine | 2 | 1.6 | 8.10E-02 | 4.20E-01 |
| positive regulation of renal sodium excretion | 2 | 1.6 | 8.10E-02 | 4.20E-01 |
| positive regulation of synaptic transmission, GABAergic | 2 | 1.6 | 8.10E-02 | 4.20E-01 |
| adenylate cyclase-activating dopamine receptor signaling pathway | 2 | 1.6 | 8.10E-02 | 4.20E-01 |
| relaxation of cardiac muscle | 2 | 1.6 | 8.10E-02 | 4.20E-01 |
| mast cell degranulation | 2 | 1.6 | 8.80E-02 | 4.40E-01 |
| cell communication by electrical coupling involved in cardiac conduction | 2 | 1.6 | 8.80E-02 | 4.40E-01 |
| prepulse inhibition | 2 | 1.6 | 8.80E-02 | 4.40E-01 |
| negative regulation of toll-like receptor signaling pathway | 2 | 1.6 | 8.80E-02 | 4.40E-01 |
| membrane depolarization during cardiac muscle cell action potential | 2 | 1.6 | 8.80E-02 | 4.40E-01 |
| lung morphogenesis | 2 | 1.6 | 8.80E-02 | 4.40E-01 |
| synaptic transmission, dopaminergic | 2 | 1.6 | 8.80E-02 | 4.40E-01 |
| maternal behavior | 2 | 1.6 | 8.80E-02 | 4.40E-01 |
| phospholipid catabolic process | 2 | 1.6 | 9.40E-02 | 4.60E-01 |
| interleukin-1-mediated signaling pathway | 2 | 1.6 | 9.40E-02 | 4.60E-01 |
| insulin-like growth factor receptor signaling pathway | 2 | 1.6 | 9.40E-02 | 4.60E-01 |
| grooming behavior | 2 | 1.6 | 9.40E-02 | 4.60E-01 |
| cellular glucose homeostasis | 2 | 1.6 | 9.40E-02 | 4.60E-01 |
